# Mechanism of Cas9 inhibition by AcrIIA11

**DOI:** 10.1093/nar/gkaf318

**Published:** 2025-04-25

**Authors:** Kaylee E Dillard, Hongshan Zhang, Lianne Z Dubbs, Chia-Wei Chou, Cynthia Terrace, Kamyab Javanmardi, Wantae Kim, Kevin J Forsberg, Ilya J Finkelstein

**Affiliations:** Department of Molecular Biosciences and Institute for Cellular and Molecular Biology, University of Texas at Austin, Austin, TX 78712, United States; Department of Molecular Biosciences and Institute for Cellular and Molecular Biology, University of Texas at Austin, Austin, TX 78712, United States; Department of Molecular Biosciences and Institute for Cellular and Molecular Biology, University of Texas at Austin, Austin, TX 78712, United States; Department of Molecular Biosciences and Institute for Cellular and Molecular Biology, University of Texas at Austin, Austin, TX 78712, United States; Department of Molecular Biosciences and Institute for Cellular and Molecular Biology, University of Texas at Austin, Austin, TX 78712, United States; Department of Molecular Biosciences and Institute for Cellular and Molecular Biology, University of Texas at Austin, Austin, TX 78712, United States; Department of Molecular Biosciences and Institute for Cellular and Molecular Biology, University of Texas at Austin, Austin, TX 78712, United States; Department of Microbiology, University of Texas Southwestern Medical Center, Dallas, TX 75390, United States; Department of Molecular Biosciences and Institute for Cellular and Molecular Biology, University of Texas at Austin, Austin, TX 78712, United States; Center for Systems and Synthetic Biology, University of Texas at Austin, Austin, TX 78712, United States

## Abstract

Mobile genetic elements evade CRISPR–Cas adaptive immunity by encoding anti-CRISPR proteins (Acrs). Acrs inactivate CRISPR–Cas systems via diverse mechanisms but generally coevolve with a narrow subset of Cas effectors that share high sequence similarity. Here, we demonstrate that AcrIIA11 inhibits *Streptococcus pyogenes* (*Sp*), *Staphylococcus aureus (Sa)*, and *Francisella novicida (Fn)* Cas9s *in vitro* and in human cells. Single-molecule imaging reveals that AcrIIA11 hinders *Sa*Cas9 target search by reducing its diffusion on nonspecific DNA. DNA cleavage is inhibited because the AcrIIA11:*Sa*Cas9 complex binds to protospacer adjacent motif (PAM)-rich off-target sites, preventing *Sa*Cas9 from reaching its target. AcrIIA11 also greatly slows down DNA cleavage after *Sa*Cas9 reaches its target site. A negative-stain electron microscopy reconstruction of an AcrIIA11:*Sa*Cas9 RNP complex reveals that the heterodimer assembles with a 1:1 stoichiometry. Physical AcrIIA11–Cas9 interactions across type IIA and IIB Cas9s correlate with nuclease inhibition and support its broad-spectrum activity. These results add a kinetic inhibition mechanism to the phage-CRISPR arms race.

## Graphical Abstract

**Graphical Abstract:**
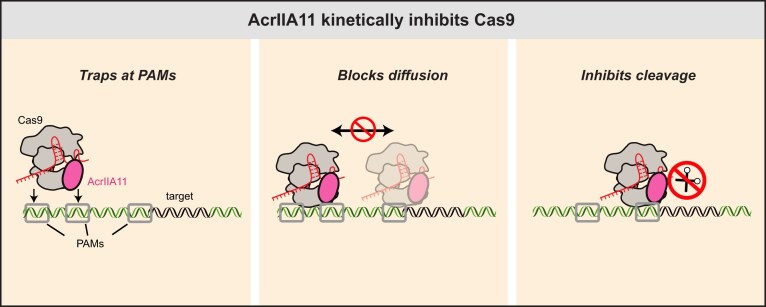

## Introduction

The molecular arms race between prokaryotes and mobile genetic elements (MGEs) has driven the evolution of anti-CRISPR proteins (Acrs) that suppress CRISPR–Cas adaptive immunity [1–4]. CRISPR–Cas systems protect bacteria and archaea from MGEs by incorporating a fragment of the foreign nucleic acid into the host genome as a spacer. This spacer is transcribed into a CRISPR RNA (crRNA) and assembled with CRISPR–Cas proteins into an effector complex. The effector complex can then target and degrade the MGEs upon later reinfection [5–8]. The first Acrs were discovered in *Pseudomonas aeruginosa* lysogens containing an active type I-F CRISPR–Cas system that was sensitive to phage plaquing [9–13]. Since this pioneering work, >100 natural Acrs have been identified via functional selections and bioinformatical approaches [14–28]. The diversity of Acr proteins also leads to different mechanisms of CRISPR–Cas suppression, including the inhibition of effector complex assembly, interference with target DNA binding, blocking of target DNA/RNA cleavage, and degradation of cyclic oligonucleotide signaling molecules [14, 29–32].

Anti-CRISPR proteins (Acrs) that inactivate class 2 Cas nucleases have emerged as important regulators for their cognate gene editors. For example, AcrIIA4, which inhibits *Sp*Cas9 by mimicking DNA, has been used to control tissue-specific gene editing [33, 34], enhance homology-directed repair during S/G2 phases [35], and improve CRISPR–Cas genome targeting precision [36]. AcrIIA4 also enables regulation of CRISPRa/CRISPRi systems in synthetic gene circuits [37]. AcrVA1, a broad-spectrum Cas12a inhibitor, acts as both a PAM mimic and an RNase that cleaves the Cas12a crRNA [20, 38]. This dual action allows AcrVA1 to effectively inhibit multiple Cas12a orthologs, including *Mb*Cas12a, *Lb*Cas12a, and *As*Cas12a [20, 38]. These mechanistic studies illuminate the evolutionary arms race between CRISPR immunity and phage counter-defenses while advancing biotechnology through enhanced control of gene editing tools [16, 17, 39–41].

We recently reported that AcrIIA11 is a potent Cas9 inhibitor with broad phylogenetic distribution [21]. However, the mechanism of AcrIIA11 inhibition is distinct from any class 2 Acr mechanism. Here, we show that AcrIIA11 inhibits three frequently used Cas9 variants—*Sa*Cas9, *Sp*Cas9, and *Fn*Cas9—both *in vitro* and in human cells. Using single-molecule imaging, we show that *Sa*Cas9 ribonucleoprotein (RNP) slide on double-stranded DNA (dsDNA) in search of the crRNA-complementary target DNA. However, AcrIIA11 restricts the one-dimensional diffusion of *Sa*Cas9 RNP, trapping it at off-target sites that are enriched for protospacer adjacent motifs (PAMs). By trapping *Sa*Cas9 at these PAM-rich off-target sites, AcrIIA11 restricts the nuclease from accessing its target. If *Sa*Cas9 pre-binds the target, AcrIIA11 also slows down cleavage of both the target and nontarget DNA strands. Negative stain electron microscopy (ns-EM) data reveals that AcrIIA11:*Sa*Cas9 is a complex with 1:1 stoichiometry. Finally, physical interactions between AcrIIA11 and Cas9 variants are crucial for its broad-spectrum kinetic inhibition of diverse type-II Cas9 orthologs. In sum, we demonstrate that AcrIIA11 is the first broad-spectrum Acr that kinetically inhibits type-II Cas9 to support phage infection.

## Materials and methods

### Protein cloning and purification

*Sa*Cas9 (Addgene #101086), *Fn*Cas9 (Addgene #130966), and AcrIIA11 were cloned into a pET19 expression vector containing an N-terminal 6xHis-TwinStrep-SUMO fusion [42, 43]. *Sa*Cas9 and *Fn*Cas9 encoded an N-terminal 3xFLAG epitope. Nuclease dead *Sa*Cas9 (d*Sa*Cas9) was created using primers KD197 and KD198 to mutate residues D10A and N580A (Supplementary Table S1). AcrIIA11 used in pull-down assays was cloned into a pET19 expression vector with either a C-terminal TwinStrep (TS) or 6xHis epitope. SUMO protease was purified as previously described [44]. For the TS–SUMO–*Sa*Cas9–sgRNA copurification with AcrIIA11, the T7 promoter and single guide RNA (sgRNA) were cloned downstream of *Sa*Cas9 in the pET19 vector (Supplementary Table S2). pMCSG7-WT- Neisseria meningitidis(*Nme*)Cas9 was a gift from Dr. Erik Sontheimer (Addgene plasmid #71474) [45]. *Fn*Cas9 protein for *in vitro* cleavage assays was purchased from Millipore Sigma (FNCAS9PROT-250UG). *Sp*Cas9 protein was purchased from NEB (M0386M).

*Sa*Cas9 was expressed in Rosetta (DE3) pLysS cells (VWR, 80509-788) and grown in 2 l of LB supplemented with 100 and 34 μg/ml carbenicillin and chloramphenicol, respectively, at 37°C to an OD_600_ ∼0.6. Cells were induced with 500 μM isopropyl β-d-1-thiogalactopyranoside (IPTG) and grown overnight (∼16 h) at 18°C. After induction, cells were pelleted via centrifugation and resuspended in *Sa*Cas9 Lysis Buffer (200 mM NaCl, 25 mM HEPES, pH 7.5, and 2 mM Dithiothreitol (DTT)) with protease inhibitors and DNase before lysing with sonication. Cellular debris was pelleted by ultracentrifugation before placing lysate over a Strep-Tactin Superflow 50% suspension (IBA Life Sciences, 2-1206-025) gravity column equilibrated in Lysis Buffer. The column was washed with 100 ml of *Sa*Cas9 Lysis Buffer and eluted with 20 ml of *Sa*Cas9 Elution Buffer (200 mM NaCl, 25 mM HEPES, pH 7.5, 5 mM desthiobiotin, and 2 mM DTT). The eluate was concentrated with a 50 kDa Amicon Ultra-15 Centrifugal Filter (Millipore Sigma, UFC905096) and incubated with SUMO protease at 4°C overnight (∼16 h). *Sa*Cas9 was isolated using a HiLoad 16/600 Superdex 200 pg column (Cytiva, 28 989 335) equilibrated in *Sa*Cas9 SEC Buffer (200 mM NaCl, 25 mM HEPES, pH 7.5, 5% glycerol, and 2 mM DTT). Peak fractions were concentrated and frozen with liquid nitrogen before storing at −80°C.

AcrIIA11 was expressed in BL21 (DE3) RIL cells and grown in either LB or TB supplemented with 100 and 34 μg/ml carbenicillin and chloramphenicol, respectively, at 37°C to an OD_600_ ∼0.6. Cells were induced with 200 μM IPTG for ∼16 h at 18°C. Cells were pelleted and resuspended in AcrIIA11 Lysis Buffer (500 mM NaCl, 25 mM HEPES, pH 7.5, and 2 mM DTT) with protease inhibitors and DNase. Cells were lysed either via sonication or via the LM10 Microfluidizer before ultracentrifugation. Clarified lysate was placed on a Strep-Tactin Superflow 50% suspension (IBA Life Sciences, 2-1206-025) gravity column equilibrated in AcrIIA11 Lysis Buffer. The column was washed with 100 ml of AcrIIA11 Lysis Buffer and the protein was eluted with 20 ml of AcrIIA11 Elution Buffer (150 mM NaCl, 25 mM HEPES, pH 7.5, 5 mM desthiobiotin, and 2 mM DTT). The protein was incubated with SUMO protease at 4°C overnight before flowing over a Ni-NTA gravity column (Thermo Fisher Scientific, 88222) to remove the cleaved 6xHis-TS-SUMO tag and SUMO Protease. The flow-through was concentrated to 1 ml using a 10 kDa Amicon Ultra-15 Centrifugal Filter (Millipore Sigma, UFC901096) before placing over a 5 ml Q column (Cytiva, 17515901) equilibrated in Q Buffer A (150 mM NaCl, 25 mM HEPES pH 7.5, 2 mM DTT, and 5% glycerol). The protein was eluted via a linear gradient with Q Buffer B (1 M NaCl, 25 mM HEPES, pH 7.5, 5% glycerol, and 2 mM DTT). Peak fractions were collected and concentrated to 1 ml and isolated on a HiLoad 16/600 Superdex 200 pg column (Cytiva, 28989335) equilibrated in AcrIIA11 SEC Buffer (200 mM NaCl, 25 mM HEPES, pH 7.5, 2 mM DTT, and 10% glycerol). Peak fractions were concentrated and frozen with liquid nitrogen before storing at −80°C.

*Nme*Cas9 was expressed in Rosetta 2(DE3) cells (Millipore Sigma, 71400-3) and grown in LB supplemented with 100 μg/ml carbenicillin and 34 μg/ml chloramphenicol. Cultures were grown at 37°C to an OD_600_ ∼0.6 and induced with 500 μM IPTG for ∼16 h at 18°C. Cells were harvested by centrifugation at 6000 × *g* for 15 min using a JLA 8.1000 rotor and resuspended in *Nme*Cas9 Lysis Buffer (500 mM NaCl, 50 mM Tris–HCl, pH 8, 10% glycerol, and 1 mM Tris(2-Chloroethyl) Phosphate (TCEP)) with protease inhibitors and DNase. Cells were lysed by sonication and pelleted using ultracentrifugation. Clarified lysate was placed on a 5 ml of Ni-NTA gravity column and washed with 100 ml of *Nme*Cas9 Wash Buffer (500 mM NaCl, 50 mM Tris–HCl, pH 8, 10% glycerol, 1 mM TCEP, and 25 mM imidazole). *Nme*Cas9 was eluted with 15 ml of NmeCas9 Elution Buffer (500 mM NaCl, 50 mM Tris–HCl, pH 8, 10% glycerol, 1 mM TCEP, and 200 mM imidazole). The protein was dialyzed overnight (∼16 h) at 4°C with TEV protease into *Nme*Cas9 Dialysis Buffer (150 mM KCl, 20 mM HEPES, pH 7.5, 5% glycerol, and 1 mM DTT). The sample was concentrated to 2 ml with a 30 kDa Amicon Ultra-15 Centrifugal Filter (Millipore Sigma, UFC903096) and placed on a 5 ml Heparin column (Cytiva, 17040703) equilibrated in *Nme*Cas9 Heparin A Buffer (150 mM KCl, 20 mM HEPES, pH 7.5, 5% glycerol, and 1 mM DTT). The protein was eluted by a linear gradient with *Nme*Cas9 Heparin B Buffer (1 M KCl, 20 mM HEPES, pH 7.5, 5% glycerol, and 1 mM DTT). The peak fractions were collected and spin concentrated to 1 ml before placing on the HiLoad 16/600 Superdex 200 pg column (Cytiva, 28989335) equilibrated in *Nme*Cas9 SEC Buffer (150 mM KCl, 20 mM HEPES, pH 7.5, 5% glycerol, and 1 mM DTT). Peak fractions were collected, spin concentrated, and frozen with liquid nitrogen before storing at −80°C.

*Fn*Cas9 was expressed in Rosetta (DE3) pLysS cells (VWR, 80509-788) and grown in 2 l of LB supplemented with 100 and 34 μg/ml carbenicillin and chloramphenicol, respectively. Cultures were grown at 37°C to an OD_600_ ∼0.6. Cells were induced with 500 μM IPTG and grown for ∼16 h at 18°C. Cells were pelleted by centrifugation at 6000 × *g* for 15 min using a JLA 8.1000 rotor and then resuspended in *Fn*Cas9 Lysis Buffer (500 mM NaCl, 25 mM HEPES, pH 7.5, 5% glycerol, and 2 mM DTT) with protease inhibitors and DNase. Cells were lysed using sonication and pelleted via ultracentrifugation. Clarified lysate was placed on a 5 ml of Strep-Tactin Superflow 50% suspension (IBA Life Sciences, 2-1206-025) gravity column equilibrated in *Fn*Cas9 Lysis Buffer. The column was washed with 100 ml of *Fn*Cas9 Lysis Buffer and eluted with 20 ml of *Fn*Cas9 Elution Buffer (200 mM NaCl, 25 mM HEPES, pH 7.5, 5% glycerol, 5 mM desthiobiotin, and 2 mM DTT). The sample was spin concentrated with a 50 kDa Amicon Ultra-15 Centrifugal Filter (Millipore Sigma, UFC905096) and incubated with SUMO protease at 4°C overnight (∼16 h). The sample was placed over a Superdex 200 Increase 10/300 GL (Cytiva, 28990944) equilibrated in *Fn*Cas9 SEC Buffer (200 mM NaCl, 25 mM HEPES, pH 7.5, 5% glycerol, and 2 mM DTT), and peak fractions were collected, spin concentrated, and frozen with liquid nitrogen before storing at −80°C.

### Human cell culture genome editing

The *Sa*Cas9 and CMV promoter-driven AcrIIA4 expression vectors were purchased from Addgene (Plasmid #85452 and #113038) [46, 47]. Previously published sgRNAs for *CACNA1D*, *EMX1*, *FANCF*, and *RUNX1* (Supplementary Table S1) were incorporated into the *Sa*Cas9 expression vector using Golden Gate cloning via Esp3I cut sites [48]. The AcrIIA4 expression vector was modified to express AcrIIA11 with a C-terminal NLS and HA tags using the HiFi Assembly Kit (NEB).

HEK293T cells were cultured in Dulbecco's Modified Eagle's Medium (DMEM) (Thermo Fisher/Gibco) containing phenol red, 4 mM L-glutamine, 110 mg/l sodium pyruvate, 4.5 g/l d-glucose, and supplemented with 10% (v/v) FBS (Thermo Fisher/Gibco) and 100 U/ml penicillin + 100 μg/ml streptomycin (Thermo Fisher/Gibco). Cell lines were tested for mycoplasma contamination via the Mycoplasma Detection Kit (Southern Biotech). Transient transfections were performed with Lipofectamine 2000 (Life Technologies). Approximately 350 000 cells were seeded in each well of a 12-well plate 24 h before transfection. Wells were transfected with either the Acr expression vector, the *Sa*Cas9/sgRNA expression vector, or both using 500 ng for each vector (3:1 Acr:*Sa*Cas9/sgRNA plasmid ratio) and either 1.5 or 3 μl of Lipofectamine (3 μl per μg of DNA).

HEK293T cells were collected and pelleted 72 h post-transfection for genomic DNA extraction using the Wizard Genomic DNA Purification Kit (Promega). The target locus was Polymerase Chain Reaction (PCR)-amplified using AccuPrime Pfx high-fidelity DNA polymerase (Thermo Fisher) and the following PCR conditions: 95°C for 2 min, 35 cycles of 98°C for 15 s + 64°C for 30 s + 68°C for 2 min, and 68°C for 2 min. Reaction-specific primers are listed in (Supplementary Table S1). Indel frequencies at the *Sa*Cas9 target site were assessed via a T7E1 assay with the EnGen Mutation Detection Kit (NEB), using the manufacturer’s recommendations. Reaction products were analyzed on a 1.3% SeaKem GTG agarose gel (Lonza) and imaged with the InGenuis3 (Syngene). For calculating indel percentages from gel images, bands from each lane were quantified with GelAnalyzer (version 2010a freeware). Peak areas for each band were measured and percentages of insertions and deletions [Indel(%)] were calculated using the formula: Indel(%) = 100 × (1 – (1 – Fraction cleaved) × 0.5), where Fraction cleaved = (Σ (Cleavage product bands))/(Σ (Cleavage product bands + PCR input band)).

### AcrIIA11:*Sa*Cas9 RNP Co-purification

Rosetta (DE3) pLysS cells containing TS–SUMO–*Sa*Cas9–sgRNA and BL21 (DE3) RIL cells containing AcrIIA11-6xHis were grown as described above. Cell pellets were lysed, separately, via microfluidizer and pelleted by ultracentrifugation. The TS–SUMO–*Sa*Cas9–sgRNA cell lysate was applied to a 5 ml of Strep-Tactin Superflow 50% suspension (IBA Life Sciences, 2-1206-025) gravity column equilibrated in Lysis Buffer containing 200 mM NaCl, 25 mM HEPES, pH 7.5, and 2 mM DTT supplemented with protease inhibitors and DNase. *Sa*Cas9 RNP was eluted with 25 ml of Elution Buffer (200 mM NaCl, 25 mM HEPES, pH 7.5, 5 mM desthiobiotin, and 2 mM DTT) and 1:100 ratio of SUMO protease was incubated with the elution at 4°C overnight. Cell lysate containing AcrIIA11-6xHis was applied to a 5 ml of Ni-NTA column. The column was washed with 50 ml of Lysis Buffer and eluted with 250 mM imidazole in the Lysis Buffer. The SaCas9 RNP and AcrIIA11 were mixed in a 1:2 molar ratio. Then, the complex was spin concentrated with a 50 kDa Amicon Ultra-15 Centrifugal Filter (Millipore Sigma, UFC905096) to ∼700 μl. The complex was applied to a Superose 6 Increase 10/300 GL (Cytiva, 29091596) equilibrated in SEC Buffer (200 mM NaCl, 25 mM HEPES, pH 7.5, 5% glycerol, and 2 mM DTT). Peak fractions were concentrated with a 50 kDa Amicon Ultra-15 Centrifugal Filter and frozen in liquid nitrogen before storing at −80°C.

### AcrIIA11:*Sa*Cas9 RNP negative stain EM

Purified *Sa*Cas9 RNP:AcrllA11 complex was diluted to a concentration of 0.02 mg/ml in SEC buffer (200 mM NaCl, 25 mM HEPES, pH 7.5, 5% glycerol, and 2 mM DTT). Samples were deposited on a CF-400-CU grid (Electron Microscopy Science) that had been plasma cleaned for 30 s on a Solarus 950 plasma cleaner (Gaten). Grids were stained with 1% uranyl acetate and imaged on the JOEL 2010F transmission electron microscope at 200 kV. Fifty-five micrographs were manually collected at a magnification of ×60 000 in 2K mode (corresponding pixel size = 3.6Å/pix) on a Gaten OneView Camera with IS software. CTF-estimation, particle picking, and 2D classification were performed on CisTEM [49]. Particles were then imported into cryoSPARC for further 2D classification and *ab initio* 3D reconstruction [50].

### *In vitro* AcrIIA11:Cas9 pulldown assays

We adapted the AcrIIA11-TS pull down assays from the previous work [21]. The binding buffer used for the pulldown assays consisted of 200 mM NaCl, 25 mM Tris–HCl, pH 7.5, and 5 mM MgCl_2_. Protein dilutions were made using this buffer. In 20 μl of binding reactions, 500 nM of Cas9 protein and 500 nM of corresponding sgRNA were incubated at room temperature for 20 min. Reactions without sgRNA were left on ice during the initial 20-min incubation. After this initial incubation, 10 μM AcrIIA11 with a C-terminal TwinStrep tag (AcrIIA11-TS) was added to the reactions, followed by another 20-min incubation at room temperature (for all reactions, not just the ones with sgRNA). 50 μl of a Streptactin slurry (IBA 2-1002-100) diluted in binding buffer was added to each binding reaction and incubated on a nutator at 4°C for 10 min. From this point onwards, all reaction were carried out in a 4°C cold room. After the 10-min incubation, the reactions were centrifuged for 2 min at 2000 rpm, and the supernatant from each reaction was discarded. The beads were washed four times by resuspending the beads in 100 μl of binding buffer, centrifuging the beads for 2 min at 2000 rpm, and by taking up the supernatant with a 200 μl of micropipette tip (Genesee Scientific: 23-150RL or 24-412) without disturbing the beads. After the fourth wash, the beads were resuspended in 40 μl of elution buffer (1× BXT buffer [22], 100 mM Tris–HCl, pH 7.5, 150 mM NaCl, 1 mM EDTA, and 50 mM biotin) and were incubated at room temperature for 15 min. Beads were spun down one for 2 min at 2000 rpm in the 4°C cold room, and 20 μl of the supernatant was carefully removed and mixed with 2× loading dye (2× Laemmli Sample buffer, Bio-Rad). Proteins were then separated by SDS–PAGE on 4%–20% Mini-PROTEAN^®^ TGX^™^ Precast Protein Gels in 1× TGS buffer, followed by staining with SimplyBlue SafeStain (Fisher Scientific, LC6065) and destaining with water.

### d*Sa*Cas9/ *Sp*Cas9 and AcrIIA11 EMSAs with DNA containing PAMs

DNA oligos with a 5′ Cy5 fluorescent label contained either 5× weak PAMs (KD203 and KD204), 1× strong (KD247 and KD248), 2× strong (KD245 and KD246), or 5× strong PAMs (KD201 and KD202) (Supplementary Table S1). Fluorescent oligos were incubated with 2× unlabeled oligos in Duplex Reaction Buffer (50 mM Tris–HCl, pH 8, and 100 mM NaCl) at 90°C for 3 min before slowly bringing to room temperature. Proteins were diluted in Dilution Buffer (200 mM NaCl, 25 mM HEPES, pH 7.5, 5% glycerol, and 2 mM DTT). 25 nM d*Sa*Cas9 was incubated with AcrIIA11 (250 nM, 500 nM, 1 μM, 2 μM, and 5 μM) in Cleavage Buffer (20 mM Tris–HCl, pH 8, 5% glycerol, 100 mM KCl, 5 mM MgCl_2_, and 1 mM DTT) at room temperature for 10 min. 25 nM sgRNA and 5 nM DNA were added to the reaction and incubated for 10 min at 37°C. Samples were placed on ice and 10 mg Orange G + 30% glycerol was added. 6% Native PAGE gels containing 5 mM MgCl_2_ and 5% glycerol were pre-run in running buffer (0.5× TBE, 5 mM MgCl_2_, and 5% glycerol) at 80 V for 30 min. Wells were cleaned and samples were loaded before running the gels at 150 V for 30 min at 4°C. Gels were imaged on the Typhoon FLA 9500 imager (Cytiva), and bands were quantified using GelAnalyzer 2010a. Electrophoretic mobility gel shift assays (EMSAs) with *Sp*Cas9 and AcrIIA11 were conducted as described above except 50 nM *Sp*Cas9 and sgRNA was used.

### *Sa*Cas9 and AcrIIA11 target and nontarget strand cleavage assays

DNA oligos were radiolabeled on the 5′ DNA end of either the target strand (TS; KD263) or nontarget strand (NTS; KD269) via T4 PNK (Supplementary Table S1). Radiolabeled oligo was mixed with a ∼4× excess unlabeled complementary oligo in Duplex Reaction Buffer (50 mM Tris–HCl, pH 8, and 100 mM NaCl) and heated to 90°C for 3 min before slowly bringing to room temperature. *Sa*Cas9 was diluted in Dilution Buffer (200 mM NaCl, 25 mM HEPES, pH 7.5, 5% glycerol, and 2 mM DTT) and incubated with SMART target sgRNA (Supplementary Table S1) in Cleavage Buffer (20 mM Tris–HCl, pH 8, 5% glycerol, 100 mM KCl, 5 mM MgCl_2_, and 1 mM DTT) for 10 min at room temperature. After the RNP was formed, 6 mM EDTA, pH 8.0 was added to the reaction for 10 min at room temperature. Next, ∼5 nM radiolabeled was added to 50 nM *Sa*Cas9 RNP in the presence of EDTA for 10 min at 37°C. 5 μM AcrIIA11 was added to the reaction and incubated for 10 min at 37°C. Finally, 10 mM MgCl_2_ was added to initiate the cleavage reaction at 37°C. Time points were taken and boiled for 3 min in loading dye [95% formamide, 20 mM EDTA, pH 8.0, 0.05% (w/v) bromophenol blue, and xylene cyanol]. A 15% Urea PAGE gel was prerun for 30 min at 120 V in 0.5× TBE running buffer. Wells were cleaned and the sample was loaded into the gel to run for 1.5 h at 80 V. Gels were dried for 2 h at 60°C and exposed overnight before imaging on the Typhoon FLA 9500 imager (Cytiva) and quantifying via GelAnalyzer 2010a.

### *In vitro* Cas9 ortholog inhibition assays

WT Cas9 (*Sa*Cas9, *Nme*Cas9, and*Fn*Cas9) and AcrIIA11 were diluted in Dilution Buffer (200 mM NaCl, 25 mM HEPES, pH 7.5, 5% glycerol, and 2 mM DTT). 50 nM of Cas9 was incubated with 5 μM AcrIIA11 and Cleavage Buffer (20 mM Tris–HCl, pH 8, 5% glycerol, 100 mM KCl, 5 mM MgCl_2_, and 1 mM DTT) for 10 min at room temperature. 50 nM sgRNA and 4 nM linearized plasmid DNA were added, and the samples were placed at 37°C. For each time point, the samples were added to Quench Buffer [4.5 mg Orange G, 10% glycerol, 0.1 mM EDTA, pH 8, 0.02% SDS, and ∼2 mg/ml proteinase K (Thermo Fisher Scientific, EO0491)] and incubated at 52°C for 30 min. Samples were run on a 1.25% agarose gel for 30 min at 120V before post-staining the gels with ethidium bromide. Gels were imaged using InGenuis3 (Syngene) and bands were quantified using GelAnalyzer 2010a.

### *In vitro* transcription of *Nme*Cas9 and *Fn*Cas9 sgRNA

gBlocks containing either the *Nme*Cas9 or *Fn*Cas9 sgRNA sequence were ordered from IDT. gBlocks were amplified with Q5 polymerase and primers that annealed to the gBlock ends (KD142, KD143, and KD144) (Supplementary Table S1). PCR products were run on a 2% agarose gel post-stained with SYBR safe stain (APExBIO, A8743). PCR products were gel extracted using a QIAquick Gel Extraction Kit (Qiagen, 28704), and samples were eluted with RNase-free water. sgRNA was *in vitro* transcribed using HiScribe T7 High Yield RNA Synthesis Kit (NEB, E2040S). Samples were incubated at 37°C for 16 h before purifying the sgRNA with Invitrogen TRIzol Reagent (Thermo Fisher Scientific, 15-596-018). *Sa*Cas9 and *Sp*Cas9 sgRNA were purchased from Synthego.

### d*Sa*Cas9 and AcrIIA11 EMSA with nontarget DNA

A gBlock (SaCas9 SMART Target, Supplementary Table S1) was amplified with primers IF365 and IF460 containing a 5′-ATTO647N (Supplementary Table S1). d*Sa*Cas9 and AcrIIA11 were diluted in Dilution Buffer (200 mM NaCl, 25 mM HEPES, pH 7.5, 5% glycerol, and 2 mM DTT). 50 nM d*Sa*Cas9 and different concentrations of AcrIIA11 (250 nM, 500 nM, 1 μM, 1.5 μM, and 2 μM) were incubated in Cleavage Buffer (20 mM Tris–HCl, pH 8, 5% glycerol, 100 mM KCl, 5 mM MgCl_2_, and 1 mM DTT) for 10 min at room temperature. 5 nM DNA and 50 nM sgRNA were added and the reactions were incubated for 10 min at 37°C. Samples were placed on ice, and 10 mg Orange G + 30% glycerol was added. 6% Native PAGE gels containing 5 mM MgCl_2_ and 5% glycerol were prerun in running buffer (0.5× TBE, 5 mM MgCl_2_, and 5% glycerol) for 30 min at 80 V. Wells were cleaned and samples were loaded into the gel before running it at 150 V for 1 h at 4°C. Gels were imaged on the Typhoon FLA 9500 imager (Cytiva) and quantified using GelAnalyzer 2010a.

### sgRNA EMSA

The sgRNA used in this EMSA is listed in Supplementary Table S1 (SMART target sgRNA) and was labeled on the 5′ end with ^32^P. To avoid dissociation of the *Sa*Cas9–sgRNA complex during the binding experiments, *Sa*Cas9–sgRNA EMSAs were prepared with an excess of *Sa*Cas9 (490 nM) incubated with a ^32^P-sgRNA (0.1 nM) and cold sgRNA (490 nM) mixture to preform the RNP. To form the RNP, *Sa*Cas9 and ^32^P-sgRNA substrates were incubated in charging buffer (10 mM Tris–HCl, pH 8, 5 mM MgCl_2_, and 0.2 mM DTT) at 25°C for 25 min. The RNP was prepared at a 100 nM effective concentration before adding AcrIIA11 (0.05, 0.1, 0.2, 0.8, 1.6, and 3.2 μM) and binding buffer (20 mM Tris–HCl, pH 8, 5% glycerol, 1 mM NaCl, and 1 mM DTT) for 30 min at 37°C. Samples were added to loading dye (1 mM Tris–HCl, pH 8, 0.1 mM EDTA, pH 8, 0.1 μg bromophenol blue, 0.1 μg xylene cyanol FF, and 5% glycerol). A 10% Native PAGE (0.5× TBE) was prerun at 80 V for 15 min in 0.5× TBE running buffer. Samples were loaded into the gel and ran at 110 V for 45 min. Gels were dried at 80°C for 2 h and exposed overnight. EMSAs were visualized by phosphorimaging using the Typhoon FLA 9500 imager (Cytiva).

### Single-molecule fluorescence microscopy and data analysis

All single-molecule imaging was performed using a Nikon Ti-E microscope in a prism-TIRF configuration equipped with a motorized stage (Prior ProScan II H117). Microfluidic flowcells were held by a custom-built stage heater to maintain experiments at 37°C. The flowcell was illuminated with a 488 nm (Coherent) laser through a quartz prism (Tower Optical Co.).

Microfluidic flowcells were prepared according to previously published protocols [51, 52]. Double-tethered DNA curtains were prepared with 40 μl of liposome stock solution (97.7% DOPC, 2.0% DOPE-mPEG2k, and 0.3% DOPE-biotin; Avanti #850375P, #880130P, and #870273P, respectively) in 960 μl of Lipids Buffer (10 mM Tris–HCl, pH 8, and 100 mM NaCl) incubated in the flowcell for 30 min. Then, 50 μg μl^-1^ of goat anti-rabbit polyclonal antibody (ICL Labs, #GGHL-15A) diluted in Lipids Buffer was incubated in the flowcell for 10 min. The flowcell was washed with Bovine Serum Albumin (BSA) Buffer (40 mM Tris–HCl, pH 8, 2 mM MgCl_2_, 1 mM DTT, 0.2 mg ml^-1^ BSA) and 1 μg l^-1^ of digoxigenin monoclonal antibody (Life Technologies, #700772) diluted in BSA buffer was injected and incubated for 10 min. Streptavidin (0.1 mg ml^-1^ diluted in BSA buffer) was injected into the flowcell for another 10 min. Finally, ∼12.5 ng μl^-1^ of the biotin- and dig-labeled DNA substrate were injected into the flowcell. The anti-rabbit antibody and digoxigenin antibody steps were omitted to prepare single-tethered DNA curtains.

To fluorescently stain DNA with YOYO-1, ∼1 nM YOYO-1 (Thermo Fisher, #Y3601), 1000 units of catalase (Millipore Sigma, #C100), 70 units of glucose oxidase (Millipore Sigma, # G2133), and 1% glucose (w/v) was injected into the flowcell at the end of the experiment.

For *Sa*Cas9 diffusion experiments, double-tethered curtains were assembled as described above. *Sa*Cas9 was diluted in dilution buffer (25 mM HEPES, pH 7.5, 200 mM NaCl, and 5% glycerol) and incubated with 2× sgRNA (SMART target sgRNA, Supplementary Table S1) in Cas9 charging buffer (50 mM Tris–HCl, pH 8, and 10 mM MgCl_2_) for 5–10 min at room temperature (∼25°C) before adding Monoclonal Anti-FLAG BioM2 antibody (Millipore Sigma, #F9291) and Qdot 705 Streptavidin Conjugate (Thermo Fisher, #Q10161MP) and placing the reaction on ice for ∼5 min. The reaction was diluted in imaging buffer (40 mM Tris–HCl, pH 8, 2 mM MgCl_2_, 1 mM DTT, 0.2 mg mL^-1^ BSA, and 5 μM biotin) with 50 mM NaCl to a final concentration of 0.5 nM *Sa*Cas9 and 1 nM sgRNA and injected on the microscope. The experiment was conducted in imaging buffer with 50 mM NaCl. For AcrIIA11:*Sa*Cas9 diffusion experiments, *Sa*Cas9 and AcrIIA11 were diluted in dilution buffer and incubated in Cas9 charging buffer for 10 min at room temperature before adding 2× sgRNA to the reaction for 5 min at room temperature. Reactions were then incubated for ∼5 min with Monoclonal Anti-FLAG BioM2 antibody and Qdot 705 Streptavidin Conjugate on ice before diluting in imaging buffer with 50 mM NaCl to a final concentration of 1 nM *Sa*Cas9, 2 nM sgRNA, and 100 nM AcrIIA11. The concentration of *Sa*Cas9 on double-tethered curtains was lowered relative to AcrIIA11:*Sa*Cas9 to limit the number of diffusing *Sa*Cas9s per DNA and make tracking individual molecules easier.

For the d*Sa*Cas9 binding distribution, single-tethered DNA curtains were assembled and d*Sa*Cas9 was diluted and incubated with sgRNA (λ target: 29.4 kb, Supplementary Table S1) as described above. d*Sa*Cas9 was diluted in imaging buffer (40 mM Tris–HCl, pH 8, 2 mM MgCl_2_, 1 mM DTT, 0.2 mg ml^-1^ BSA, and 5 μM biotin) with 100 mM NaCl to a final concentration of 1 nM d*Sa*Cas9 and 2 nM sgRNA. d*Sa*Cas9 RNP was injected onto the flowcell and incubated on the microscope for 5 min before imaging. The experiment was conducted in imaging buffer with 100 mM NaCl. For the AcrIIA11:d*Sa*Cas9 binding histogram, d*Sa*Cas9, AcrIIA11, and sgRNA were diluted and incubated as described above and diluted in imaging buffer with 100 mM NaCl to a final concentration of 1 nM d*Sa*Cas9, 2 nM sgRNA, and 100 nM AcrIIA11. AcrIIA11:d*Sa*Cas9 was injected into the flowcell and incubated on the microscope for 5 min before imaging.

To determine the localization and cleavage rate of WT *Sa*Cas9, single-tethered curtains were assembled. *Sa*Cas9 was diluted and incubated with sgRNA (λ target: 29.4 kb, Supplementary Table S1) as described above. *Sa*Cas9 RNP was diluted in imaging buffer (40 mM Tris–HCl, pH 8, 2 mM MgCl_2_, 1 mM DTT, 0.2 mg ml^-1^ BSA, and 5 μM biotin) with 50 mM NaCl to a final concentration of 2 nM *Sa*Cas9 and 4 nM sgRNA and injected on the microscope. AcrIIA11:*Sa*Cas9 was diluted and incubated with sgRNA as described above. The complex was diluted in imaging buffer with 50 mM NaCl to a final concentration of 2 nM *Sa*Cas9, 4 nM sgRNA, and 200 nM AcrIIA11.

A molecule was considered stationary if it stayed within a 3 × 3 pixel region of interest (ROI) around its own starting position. Molecules counted in the analysis had to bind DNA for ∼5 s or longer. The position along the DNA was determined by measuring the distance of *Sa*Cas9 relative to the barrier. The position of WT *Sa*Cas9 with and without AcrIIA11 was determined 90 s after the molecules entered the flowcell. To determine if a DNA molecule was cleaved by *Sa*Cas9, *Sa*Cas9 had to bind the target and release the DNA (loss of *Sa*Cas9 signal). The DNA was stained at the end of the experiment with YOYO-1 to confirm it was cleaved. *Sa*Cas9 molecules that bound the target and did not cleave the DNA had to remain on the DNA for the rest of the movie without cleaving it to be counted in the cleavage analysis. Molecules that bound the target and dissociated during the movie without cleaving it were not counted. Binding lifetimes were fit to a single exponential decay using a custom MATLAB script (Mathworks R2017a).

Particle trajectories were tracked using a custom ImageJ script. Mean squared displacements (MSDs) was calculated for the first 10 time intervals of each particle and fit to a line to obtain the diffusion coefficient, as previously described [53]. The mean diffusion coefficient was obtained from >30 molecules, and the standard error of the mean (S.E.M.) was determined.

## Results

### AcrIIA11 inhibits DNA cleavage by *Sa*Cas9 *in vitro* and in human HEK293T cells

**Figure 1.**
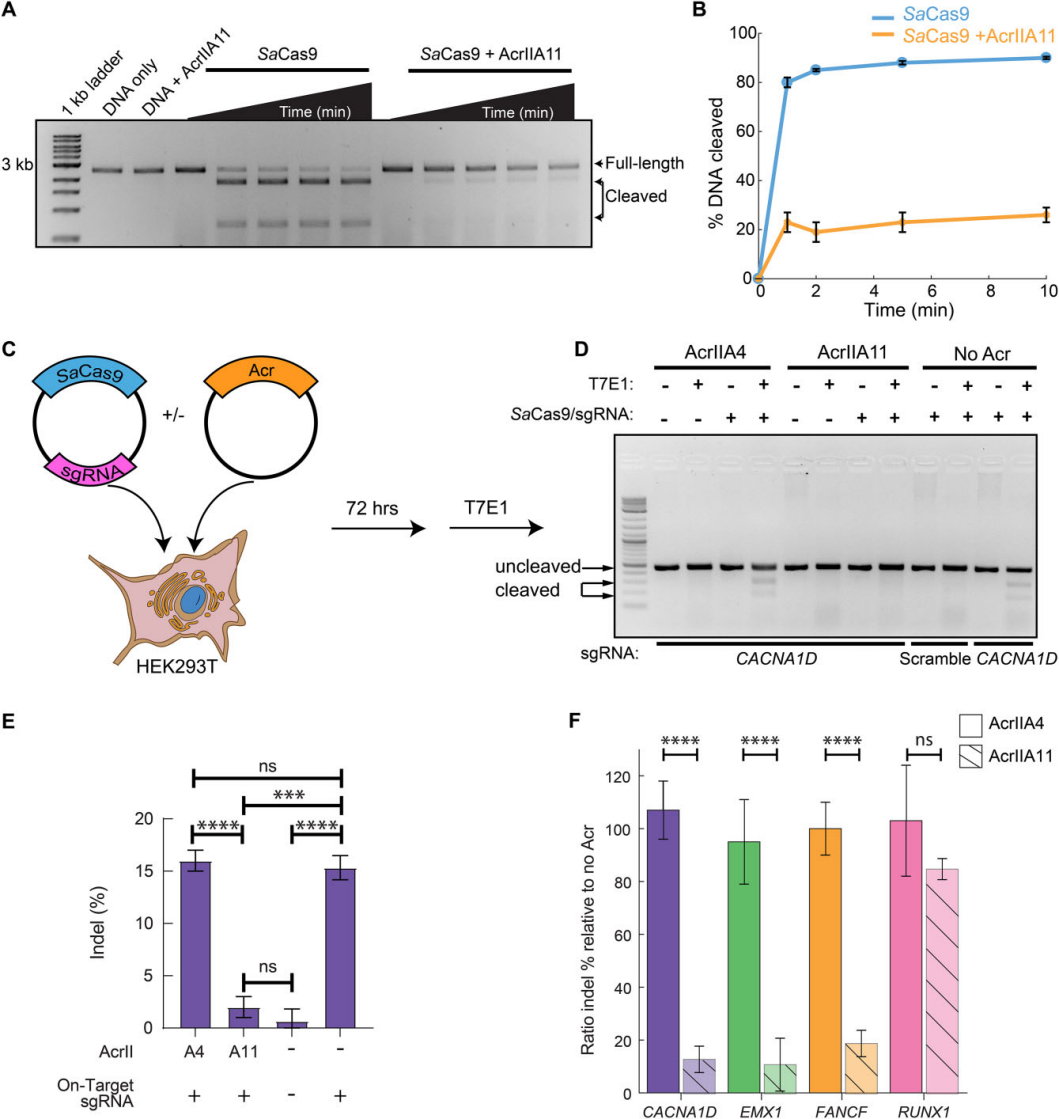
AcrIIA11 inhibits SaCas9 *in vitro* and in human HEK293T cells. (**A**) Agarose gel and (**B**) quantification of DNA cleavage by SaCas9. Graphs represent the mean of three replicates. Error bars: S.E.M. (**C**) HEK293Ts are transiently transfected with plasmids carrying SaCas9 + sgRNA. A second plasmid encodes either AcrIIA4 (positive control) or AcrIIA11. (**D**) Representative agarose gel and (**E**) quantification of indel percentage for the CACNA1D site. Error bars are the standard deviation of three replicates. *P*-values (not significant [ns], *P* > 0.05; ***P < 0.001; ****P < 0.0001) were determined using a Student’s *t*-test. (**F**) Quantification of the editing efficiency when AcrIIA11 or AcrIIA4 are present relative to when no Acr is expressed. Error bars are the standard deviation of three replicates. *P*-values (not significant [ns], *P* > 0.05; ****P < 0.0001) were determined using a Student’s *t*-test.

Previously, we showed that AcrIIA11 inhibit *Sp*Cas9 (type II-A, 1 368 amino acids) [21]. To determine the mechanisms of Cas9 inhibition, we assayed whether AcrIIA11 can inhibit DNA cleavage by other Cas9 orthologs. We focused initially on *Sa*Cas9 because it’s relatively small (1053 amino acids) and widely used in diverse genome editing applications but only shares ∼17% amino acid identity with *Sp*Cas9 [54, 55]. We first incubated AcrIIA11 with *Sa*Cas9 before adding a sgRNA and a linearized plasmid containing the target DNA adjacent to the *Sa*Cas9-specific PAM. Cleavage products were resolved on an agarose gel at various timepoints. AcrIIA11 efficiently inhibited DNA cleavage (Fig. 1A and B) via a mechanism that does not involve sgRNA cleavage (Supplementary Fig. S1).

Next, we tested whether AcrIIA11 can inhibit *Sa*Cas9 in human cells. We targeted the *CACNA1D*, *EMX1*, *FANCF*, and *RUNX1* genomic loci in HEK293T cells because these sites support robust gene editing with *Sa*Cas9 [56–59]. HEK293Ts were cotransfected with two plasmids. The first plasmid expressed *Sa*Cas9 along with a sgRNA; the second expressed AcrIIA11 or AcrIIA4 (Fig. 1C–F and Supplementary Fig. S2). AcrIIA4 was included as a negative control because it inhibits *Sp*Cas9 but not *Sa*Cas9 [60]. As expected, we did not see any cleavage when a scrambled sgRNA was used in the absence of any inhibitors or with AcrIIA4 and an on-target sgRNA. AcrIIA11 inhibited *Sa*Cas9 at the *CACNA1D, EMX1*, and *FANCF* loci (Fig. 1C–F and Supplementary Fig. S2A–D). Surprisingly, it only weakly inhibited cleavage at *RUNX1* (Fig. 1F, and Supplementary Fig. S2E and F). Of the four loci tested in here, *RUNX1* has higher cleavage activity than all other targets, as measured by the overall indel percentage. We speculate that this site is more accessible to *Sa*Cas9 and that AcrIIA11 has a shorter time window to inhibit the enzyme before a DNA break occurs. Together, these data indicate AcrIIA11 inhibits *Sa*Cas9 *in vitro* and is a locus-specific inhibitor in cells.

### AcrIIA11 inhibits the *Sa*Cas9 target search

**Figure 2.**
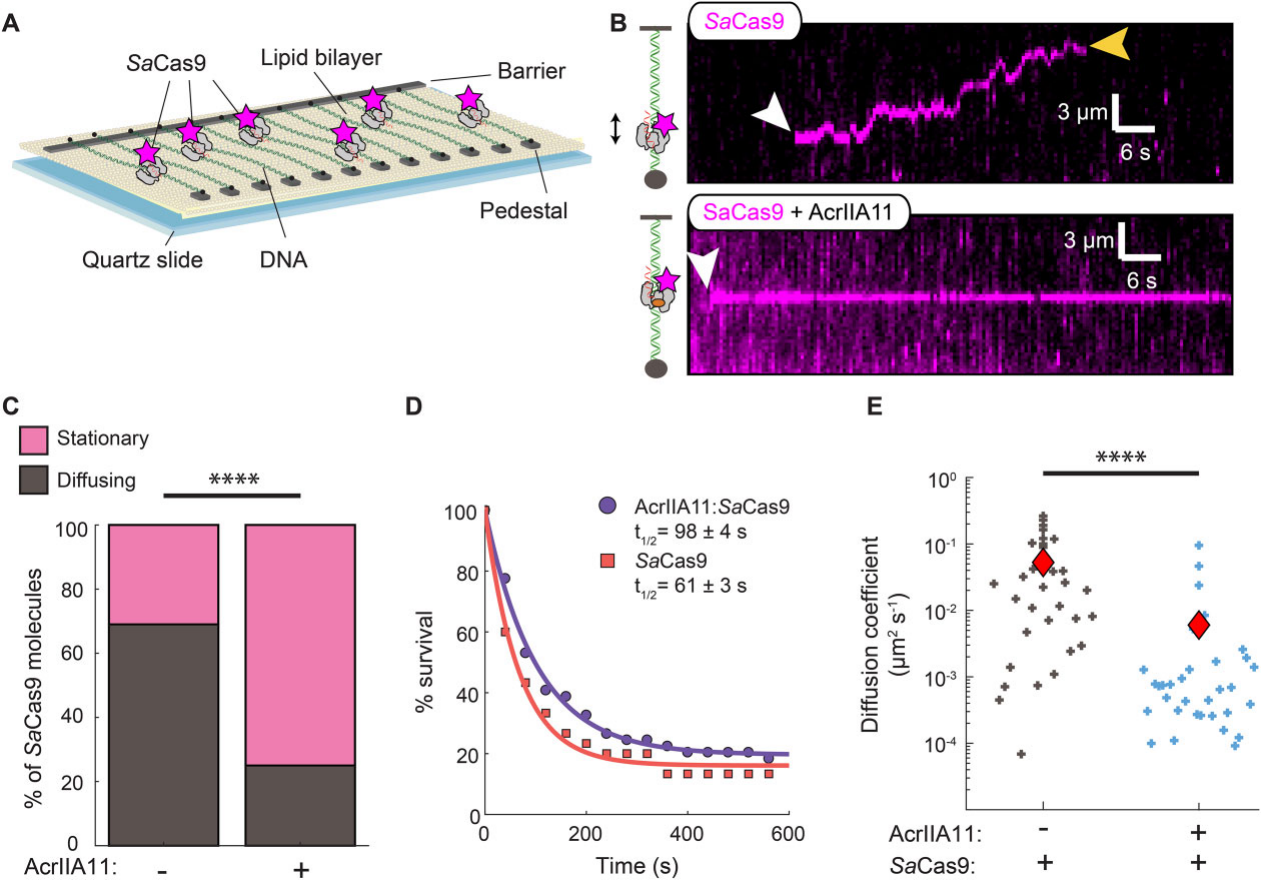
AcrIIA11 inhibits SaCas9 dynamic target searching by hindering its diffusion. (**A**) Schematic of the double-tethered DNA curtains assay. Buffer flow is turned off after both ends of the DNA are tethered between the chromium barriers and pedestals and the protein enters the flowcell. (**B**) Kymographs showing diffusing SaCas9 RNPs (top) and stationary AcrIIA11:SaCas9 RNP complexes (bottom). The white arrow indicates the time when SaCas9 binds, and the yellow arrow indicates when SaCas9 releases the DNA. (**C**) Most SaCas9 RNPs diffuse without AcrIIA11 (*N* = 89) but are stationary with AcrIIA11 (*N* = 93). *P*-value was determined by a Chi-Squared test (****P < 0.0001). (**D**) Lifetime of SaCas9 (*N* = 30) and AcrIIA11:SaCas9 (*N* = 49) on nonspecific DNA. Half-lives are indicated for each curve. (**E**) Diffusion coefficients of SaCas9 with (*N* = 33) and without (*N* = 33) AcrIIA11. *P*-value was determined by a Mann–Whitney *U*-test (****P < 0.0001). Red diamonds indicate the mean diffusion coefficient.

To understand how AcrIIA11 inhibits *Sa*Cas9, we first investigated the impact of AcrIIA11 on *Sa*Cas9 RNPs’ target binding via single-molecule imaging (Fig. 2A). In this DNA curtains assay, a 48.5 kb-long DNA substrate is suspended above a lipid bilayer between two microfabricated chromium features [51, 52]. The DNA is prepared with a biotin on one end and a digoxigenin on the opposite end. The biotinylated end is immobilized on the surface of a fluid lipid bilayer via a biotin–streptavidin linkage. In addition to capturing one end of the DNA substrate, the bilayer also passivates the flowcell surface. Next, the DNA molecules are organized and extended at microfabricated chrome barriers via buffer flow. The second DNA end is captured at the anti-digoxigenin-functionalized chrome pedestal and buffer flow is terminated. Our attempts to fluorescently label AcrIIA11 resulted in a partial loss of *Sa*Cas9 inhibition. Therefore, we fluorescently labeled *Sa*Cas9 via a fluorescent anti-FLAG antibody that targeted a 3xFLAG epitope on its N-terminus. Using the dual-tethered DNA molecules in the absence of buffer flow, we can thus track the dynamic behavior fluorescently labeled *Sa*Cas9 RNPs.

*Sa*Cas9 diffuses on nonspecific DNA as it searches for the target site, as has been reported for other CRISPR–Cas effectors [61–63]. To determine the diffusive properties of *Sa*Cas9 RNPs, we prepared the complex with a scrambled sgRNA that did not have a target in this DNA substrate. Most *Sa*Cas9 RNPs (69%; *N* = 61/89) scan the DNA via one-dimensional (1D) diffusion (Fig. 2B). The remaining 31% of the molecules appeared stationary on DNA. *Sa*Cas9 RNPs are more diffusive than *Sp*Cas9 RNPs, which only scans short stretches of DNA via 1D-diffusion [62]. Half of the *Sa*Cas9 RNPs dissociated from the DNA within 61 ± 3 s (*N* = 30). For the AcrIIA11:*Sa*Cas9 complexes, we preincubated AcrIIA11 with fluorescently labeled *Sa*Cas9 RNPs and injected the complexes into the same DNA curtain flowcells. Surprisingly, 75% of the molecules appeared stationary (*N* = 70/93) (Fig. 2C and Supplementary Table S3). AcrIIA11 also increased the lifetime of *Sa*Cas9 RNPs on nonspecific DNA to 98 ± 4 s (50% increase, *N* = 49) (Fig. 2D). For the 25% of the molecules that remained diffusive, AcrIIA11 decreased the diffusion coefficient nearly tenfold. *Sa*Cas9 RNPs had a diffusion coefficient of 0.05 ± 0.01 μm^2^ s^−1^ (mean ± S.E.M.; *N* = 33), whereas the AcrIIA11:*Sa*Cas9 complexes had a mean diffusion coefficient of 0.006 ± 0.003 μm^2^ s^−1^ (*N* = 33) (Fig. 2E and Supplementary Table S3). These results demonstrate that AcrIIA11 inhibits *Sa*Cas9 target binding by inhibiting the diffusion of the RNP on nonspecific DNA.

### PAM-rich sites trap *Sa*Cas9:AcrIIA11 complexes

**Figure 3.**
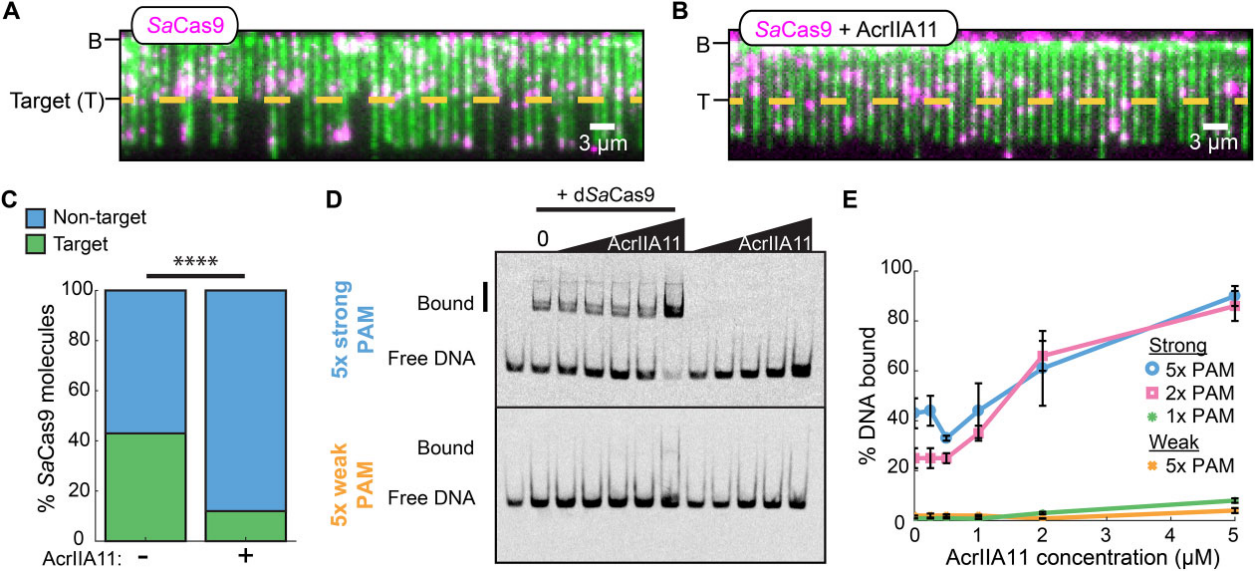
AcrIIA11 promotes the binding of SaCas9 to off-target sites with PAM enrichment. (**A**) Image of single-tethered DNA molecules after injection of SaCas9 RNPs. The short DNAs are resulted from the cleavage by SaCas9 RNPs. The target site (T) is indicated with a yellow dotted line. DNA is stained with a fluorescent intercalating dye YOYO-1 after incubation with SaCas9. (**B**) The single-tethered DNA molecules after injection of AcrIIA11:SaCas9 complexes. (**C**) Percentage of SaCas9 binding at the target with (*N* = 108) and without AcrIIA11 (*N* = 89). *P*-value was determined by a Chi-Squared test (****P < 0.0001). (**D**) Representative EMSAs of the strong 5× PAM and weak 5× PAM DNA substrates with dSaCas9 and AcrIIA11. (**E**) Quantification of weak PAM, 1×, 2×, and 5× strong PAM EMSAs. Error bars: S.E.M. of three replicates.

To determine how AcrIIA11 traps *Sa*Cas9 on nonspecific DNA, we first imaged RNPs that targeted a single site in the 48.5 kb DNA substrate (Fig. 3A and B). To visualize target binding and cleavage, the DNA molecules were tethered to the flowcell via a single DNA end and fluorescently labeled with the intercalating dye after *Sa*Cas9 injection. Injecting 2 nM *Sa*Cas9 RNPs resulted in 43% (*N* = 38/89) of the molecules binding at the target site within 90 s of the RNP entering the flowcell (Fig. 3C and Supplementary Table S3). Most *Sa*Cas9 RNPs that did not localize to the target slid toward the free DNA end, consistent with buffer flow-induced biased diffusion (Supplementary Fig. S3A). Preincubating *Sa*Cas9 RNPs with AcrIIA11 resulted drastically decreased target-bound RNPs (12% target bound *N* = 13/108 *Sa*Cas9 RNPs) (Fig. 3C and Supplementary Table S3). Only 19% (*N* = 18/95) of the AcrIIA11-bound RNPs slid to the DNA end, indicating that they were bound tightly to the nonspecific DNA. Similarly, nuclease dead (d*Sa*Cas9) RNPs bound the target in the absence of AcrIIA11 but bound nonspecific DNA with AcrIIA11 (Supplementary Fig. S3B–D). We confirmed these results using EMSAs (Supplementary Fig. S4A). As expected, d*Sa*Cas9 weakly bound noncomplementary DNA (11 ± 2% DNA bound) but had a higher affinity for the complementary DNA (52 ± 6% DNA bound). Increasing concentrations of AcrIIA11 increased d*Sa*Cas9 binding to the noncomplementary DNA up to ∼3-fold (32 ± 3% bound DNA at 2 μM AcrIIA11) (Supplementary Fig. S4B). Together, the single-molecule and ensemble biochemistry results indicate that AcrIIA11 increases off-target binding of *Sa*Cas9 RNPs to inhibit RNP diffusion and prevent target recognition.

We hypothesized that AcrIIA11 traps *Sa*Cas9 RNPs on PAM-rich off-target sites. To examine this, we performed an EMSA assay in which dSaCas9 was pre-incubated with AcrIIA11 before adding sgRNA and nontarget dsDNA labeled at the 5′ end with a Cy5 fluorophore. The dsDNA contained one, two, or five high-affinity PAMs (5′-NNGGGT), or five low-affinity PAM (5′-NNTCTCN) while maintaining overall GC% bias [55]. In contrast to *Sa*Cas9 RNPs, the AcrIIA11:*Sa*Cas9 strongly preferred PAM-rich DNA (Fig. 3D and E, and Supplementary Fig. S5A). The binding of AcrIIA11:d*Sa*Cas9 increased with the number of PAMs, with 90% of the 5× strong PAM DNA bound at the highest AcrIIA11 concentration. There was a noticeable increase in AcrIIA11:d*Sa*Cas9 binding when transitioning from a single PAM to two PAMs. Consistent with observations for *Sa*Cas9, AcrIIA11 also increased nonspecific *Sp*Cas9 binding to a DNA substrate containing five strong PAMs, but not to a DNA with five weak PAMs (Supplementary Fig. S5B and C). Taken together, these results show that AcrIIA11 inhibits RNP target search kinetics by trapping the enzyme at PAM-rich off-target sites.

### AcrIIA11 inhibits DNA cleavage at preformed complexes

**Figure 4.**
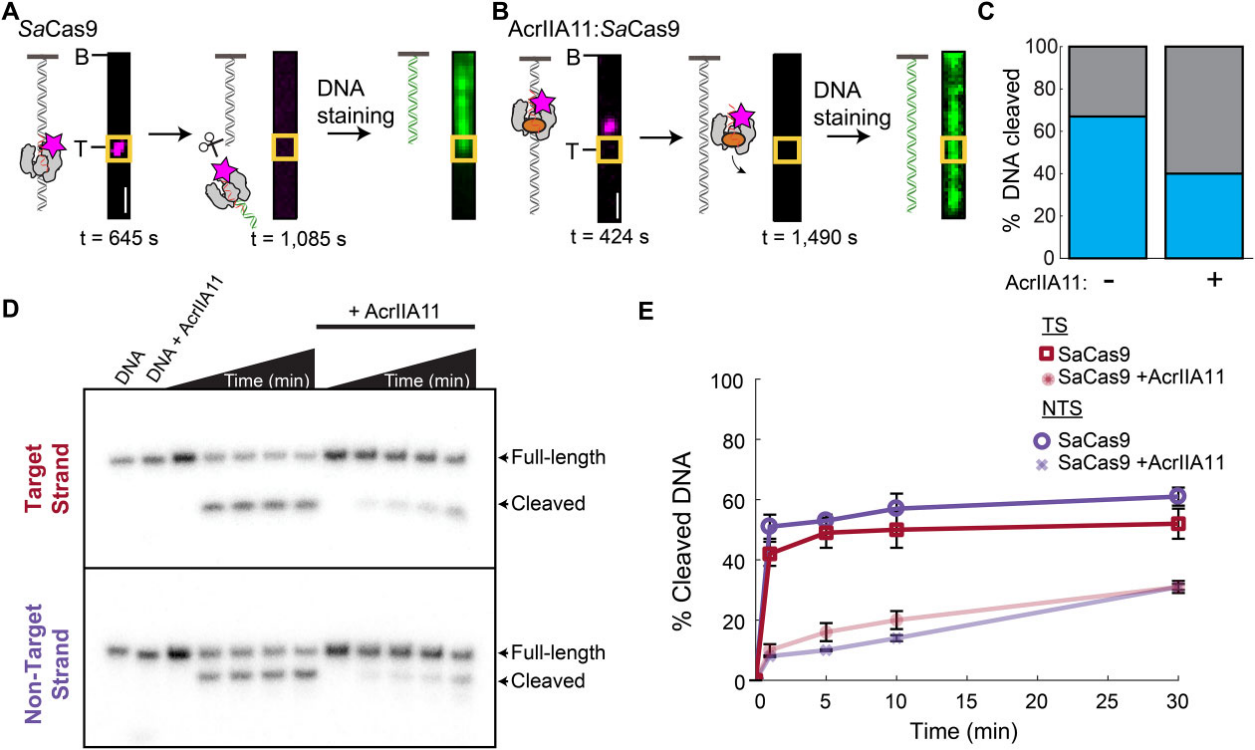
AcrIIA11 inhibits but does not abolish SaCas9 on target cleavage activity. (**A**) Schematic and images of SaCas9 binding (left), cleaving/ releasing the DNA (middle), and the cleaved DNA (right). The target site is labeled with a yellow box. Scale bar: 3 μm. (**B**) Schematic and images of AcrIIA11:SaCas9 binding at an off-target site on the DNA (left), dissociating from the DNA (middle), and the remaining full-length DNA (right). The target site is labeled with a yellow box. Scale bar: 3 μm. (**C**) Percentage of DNA cleavage by SaCas9 at the target with (*N* = 10) and without AcrIIA11 (*N* = 30). (**D**) Representative SaCas9 cleavage gels with and without AcrIIA11. The annealed DNA substrate has either a 5′radiolabel on the TS (top) or NTS (bottom). (**E**) Quantification of SaCas9 cleavage with and without AcrIIA11 for the target and nontarget DNA strands. Error bars: S.E.M. of three replicates.

To determine whether AcrIIA11 inhibits DNA cleavage, we first analyzed AcrIIA11:*Sa*Cas9 complexes that bound the target site in the single-molecule assay described above. *Sa*Cas9 is a multiple turnover enzyme that releases the PAM distal DNA after cleavage [64, 65]. We designed the target DNA sequence so that *Sa*Cas9-catalyzed cleavage released both the enzyme and the DNA fragment from the tethered DNA end. Cleavage reactions could thus be determined by (i) *Sa*Cas9 binding to the target site, (ii) subsequent loss of the fluorescent *Sa*Cas9 signal, and (iii) DNA cleavage at the target site, as confirmed by fluorescence staining and imaging of the remaining tethered DNA (Fig. 4A and B). *Sa*Cas9 RNPs that stably bound the target cleaved and released 67% (*N* = 20/30) of the DNA molecules (as imaged ∼15 min after RNP injection) (Fig. 4A). AcrIIA11:*Sa*Cas9 complexes that bound off-target sites dissociated without cleavage (Fig. 4B). We rarely observed AcrIIA11:*Sa*Cas9 complexes at the target DNA but 40% (*N* = 4/10) of those at the target site cleaved the DNA (Fig. 4C). The single-molecule results indicate that AcrIIA11 inhibits *Sa*Cas9 RNPs’ cleavage activity.

To further assess whether AcrIIA11 blocks *Sa*Cas9 cleavage, we preincubated the enzyme with its target DNA in the absence of MgCl_2_ to form the R-loop. When MgCl_2_ was added to the reaction, *Sa*Cas9 RNPs efficiently cleaved the TS and NTS (Fig. 4D and E). In contrast, adding AcrIIA11 before MgCl_2_ significantly inhibited both TS and NTS cleavage. Together, the single-molecule and ensemble results demonstrate that AcrIIA11 inhibits but doesn’t completely abolish target cleavage with prebound Cas9 RNPs.

### *Sa*Cas9 and AcrIIA11 form a complex with 1:1 stoichiometry

**Figure 5.**
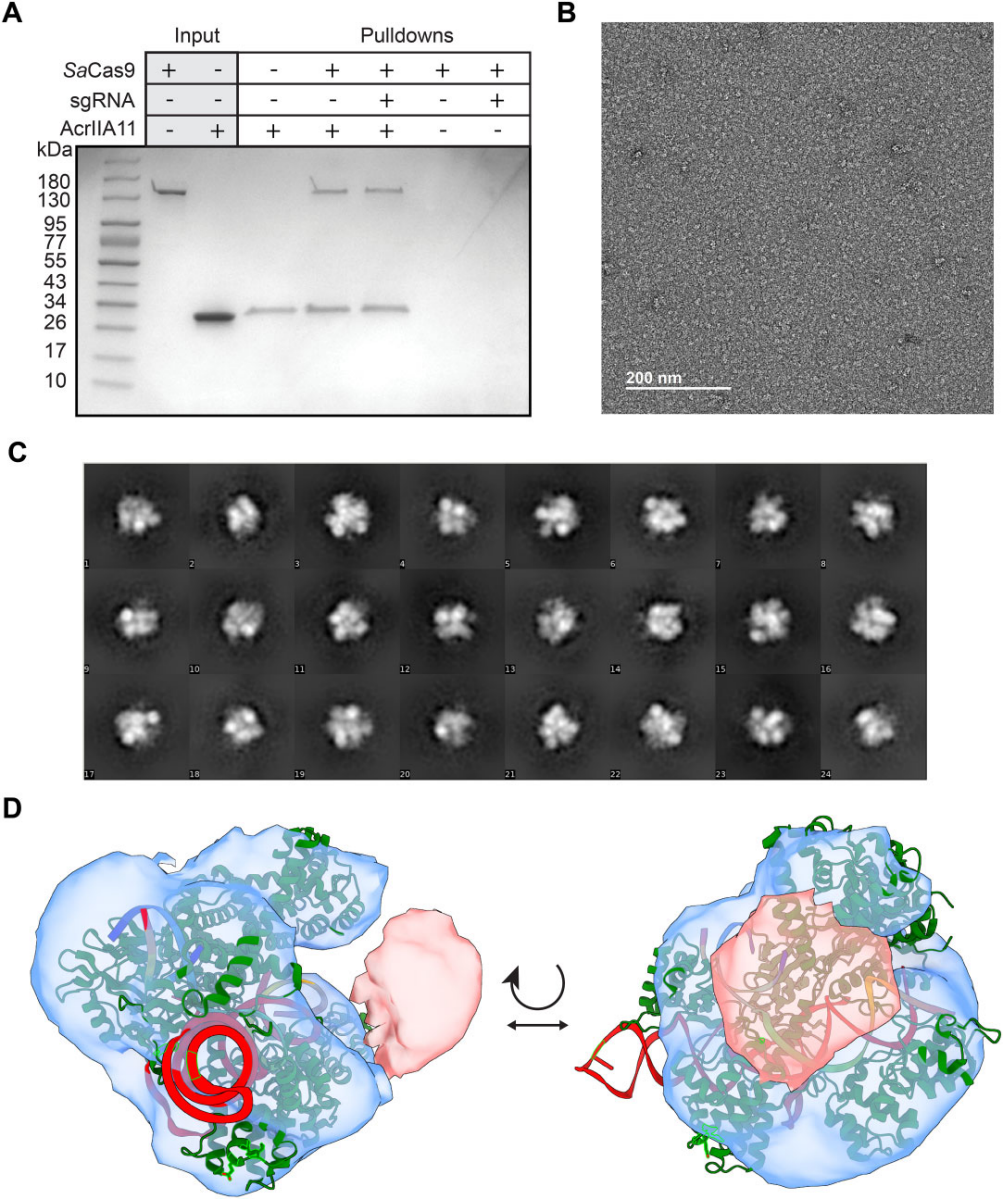
AcrIIA11 physically binds SaCas9 to form a AcrIIA11:SaCas9 complex. (**A**) Coomassie-stained SDS–PAGE gel of Step-Tactin immobilized AcrIIA11 pulldown of SaCas9. (**B**) Negative stain electron microscope grid of SaCas9 RNP and AcrIIA11. (**C**) Representative 2D averages. (**D**) 3D model of AcrIIA11:SaCas9 with a crystal structure of SaCas9 RNP docked into the 3D model (green, PDB: 5CZZ). Extra density in pink is believed to be AcrIIA11.

We previously showed that AcrIIA11 physically interacts with *Sp*Cas9 [21]. Based on these results, we speculated that the physical interaction between AcrIIA11 and Cas9 orthologs is necessary for its broad inhibition mechanism. We first confirmed that AcrIIA11 also binds *Sa*Cas9 (Fig. 5A). In these experiments, AcrIIA11 with a C-terminal TwinStrep epitope was immobilized on Strep-tactin resin and incubated with either apoCas9 or the RNP. The complex was then eluted from the resin and analyzed on an SDS–PAGE gel. Resin-immobilized AcrIIA11 captured *Sa*Cas9 with or without its sgRNA (Fig. 5A). AcrIIA11 also interacted with and inhibited *Fn*Cas9 (type II-B, 1629 amino acids), one of the largest Cas9 orthologs that is frequently used for gene editing and CRISPR diagnostics [66] (Supplementary Fig. S6). However, AcrIIA11 does not bind or inhibit *Nme*Cas9 (type II-C, 1082 amino acids), which only shares 27% sequence identity with *Sa*Cas9 [67] (Supplementary Fig. S6). These results underscore the importance of physical interactions between AcrIIA11 and type-II Cas9 orthologs for Acr activity.

We next characterized the organization of the AcrIIA11:*Sa*Cas9 RNP complex via negative-stained electron microscopy (Fig. 5B–D). To assemble the complex, we incubated *Sa*Cas9 RNP with a ∼2-fold stoichiometric excess of AcrIIA11. This mixture was passed over a gel filtration column prior to deposition on EM grids for imaging (Supplementary Fig. S6G). This procedure yielded a peak that eluted at a higher molecular weight than the *Sa*Cas9 RNP. The resulting complexes were monodispersed when imaged on the EM grids (Fig. 5B). We modelled the *Sa*Cas9 RNP structure into the low-resolution three-dimensional model that we obtained from 2D class averaging of 16 761 particles (Fig. 5C). Additional density that likely corresponds to AcrIIA11 was evident near the HNH domain and part of the RuvC domain of *Sa*Cas9 (Fig. 5D). An AlphaFold-generated model of the AcrIIA11 monomer could be accommodated into this density without obvious steric clashes. Although we weren’t successful in obtaining a higher-resolution cryo-EM structure, these data indicate that the AcrIIA11:*Sa*Cas9 has a 1:1 stoichiometry.

## Discussion

**Figure 6.**
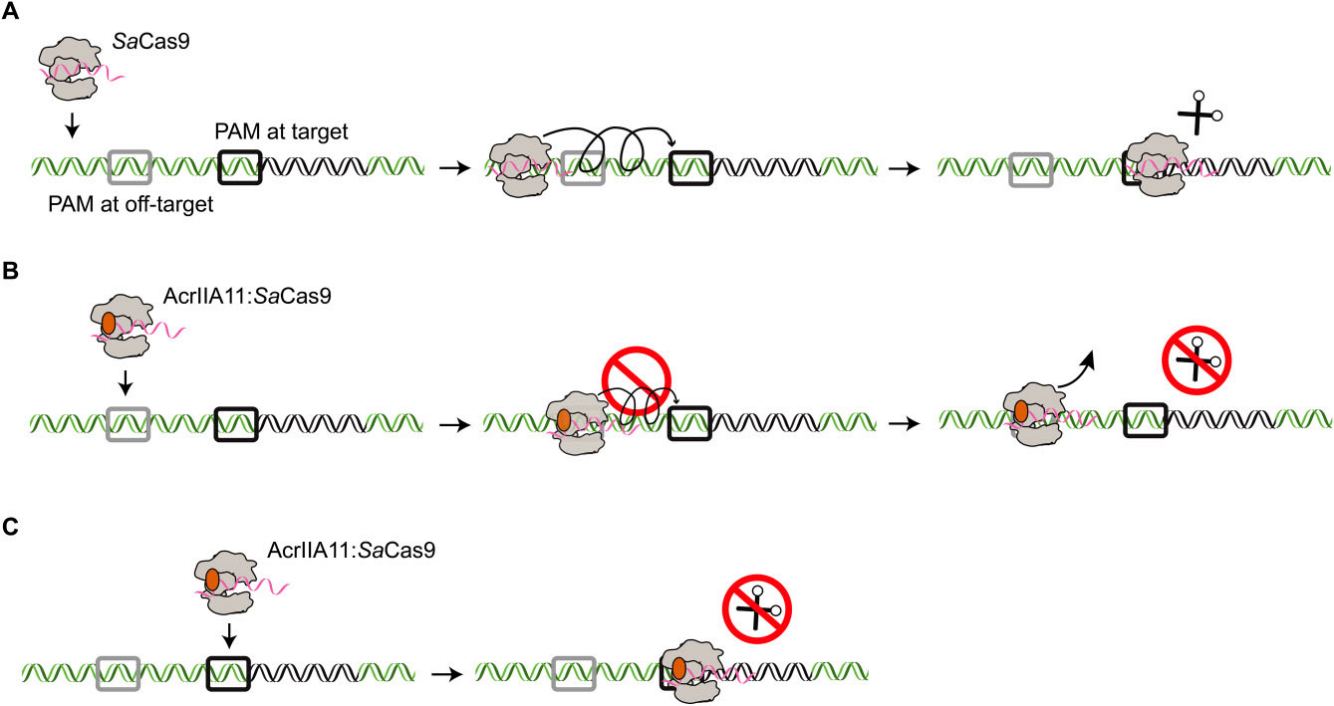
AcrIIA11 inhibition mechanism. (**A**) Cas9 initially binds off-target DNA and samples PAMs (gray) in search of the PAM (black square) that is adjacent to the target site. Next, Cas9 forms an R-loop and cleaves the target DNA. (**B**) AcrIIA11 forms a 1:1 complex with Cas9. The complex binds PAM-rich off-target sites and prevents SaCas9 diffusion and target search. (**C**) AcrIIA11 also inhibits nuclease activity for those Cas9s that found the target.

Using single-molecule and ensemble biochemical assays, we discovered a novel mechanism of Cas9 inhibition through kinetic trapping by an anti-CRISPR protein (Fig. 6). The AcrIIA11:Cas9 complex promotes binding at PAM-rich off-target sites to impede the diffusion of and target binding by Cas9 RNPs (Fig. 6). AcrIIA11 independently binds DNA with low affinity [21]. The intrinsic DNA-binding affinity of AcrIIA11 likely causes the suppressed DNA diffusion and nonspecific DNA binding by the RNP:AcrIIA11 complex. AcrIIA11 also inhibits *Sa*Cas9 cleavage after *Sa*Cas9 binds to its target (Fig. 6C). Negative-stained EM reconstructions show a 1:1 stoichiometry for the AcrIIA11:SaCas9 complex. This binding may induce conformational changes in either or both proteins. Future high-resolution structures will help elucidate the structural rearrangements that drive increased nonspecific DNA-binding and cleavage inhibition.

While this work was under review, another group reported that AcrIF9 also sequesters a type I-F Cascade complex on nonspecific DNA [68, 69]. AcrIF9 also binds its target Csy surveillance complex to promote nontarget dsDNA binding, sequestering the complex away from its intended targets. However, AcrIF9 primarily functions by sterically blocking target DNA hybridization to the crRNA guide. Furthermore, AcrIIA11 possesses intrinsic DNA affinity and specifically traps Cas9 RNPs at PAM-rich sites. This differs from the AcrIF9-Csy complex, which binds nonspecific DNA without any PAM preference. Despite these differences, the discovery that distinct anti-CRISPRs have evolved a nonspecific DNA sequestration mechanism underscores that kinetic inhibition as an effective strategy against diverse CRISPR–Cas systems. More broadly, coupling a weak DNA-binding peptide with a CRISPR-interacting domain may be sufficient to generate synthetic CRISPR–Cas inhibitors.

AcrIIA11 sequesters *Sa*Cas9 at PAM-rich off-target sites. With the short substrates tested here, two PAMs were sufficient to trap AcrIIA11:Cas9 RNPs on the nonspecific sites. Each *Sp*Cas9 searches *Escherichia coli* for up to ∼6 h to find its target, as it must query PAMs individually to check for target complementarity [70]. By increasing Cas9 residence times at the many off-target sites in both the host and invading MGE genomes, we proposed that AcrIIA11 delays CRISPR-based immunity to allow the MGE to replicate and overwhelm its host.

AcrIIA11 also inhibits *Sa*Cas9 in three of four genomic loci in HEK293T cells. However, AcrIIA11 only partially inhibited *Sa*Cas9 at *RUNX1* and *Sp*Cas9 at *EMX1* loci [21]. Our biochemical mechanism hints at a possible reason for site-specific inhibition. We propose that these sites are more accessible to the nuclease and are thus cleaved before sufficient AcrIIA11 is expressed to trap all *Sa*Cas9 molecules. Indeed, the overall indel percentage was highest at *RUNX1* compared to the other loci, suggesting that this site is highly accessible to *Sa*Cas9. Alternatively, the AcrIIA11 and *Sa*Cas9 may exist in a dynamic equilibrium where some of the complexes dissociate to transiently unleash the nuclease. Adjusting the timing of AcrIIA11 expression, the concentration of AcrIIA11 relative to SaCas9, or other Cas9 ortholog used could potentially overcome this site-specific inhibition.

Acrs that inhibit multiple Cas9 orthologs can be used as master inhibitors of Cas9 genome editing, making them versatile for potential biotechnological applications [16, 17, 39–41]. AcrIIA11 physically binds and inhibits *Sa*Cas9 (type II-A), *Sp*Cas9 (type II-A), and*Fn*Cas9 (type II-B), but not *Nme*Cas9 (type II-C) [21]. Other broad Cas9 inhibitors (AcrIIA5, AcrIIA16, and AcrIIA17) either cleave the sgRNA or alter sgRNA expression levels, resulting in irreversible Cas9 inhibition [71–73]. By contrast, AcrIIA11 inhibition is nonenzymatic; physical interactions between AcrIIA11 and multiple Cas9s suggests that AcrIIA11 recognizes a conserved pocket on the nuclease surface. Further structural analyses of the Cas9–AcrIIA11 interface and AcrIIA11–DNA interactions will facilitate designer inhibitors that act as fine-tuned on-/off- switches for Cas9-based gene editing. More broadly, kinetic trapping on nonspecific targets may be a general approach for developing inhibitors of DNA- and RNA-editing enzymes.

## Supplementary Material

gkaf318_Supplemental_File

## Data Availability

Requests for materials should be addressed to I.J.F. (ilya@finkelsteinlab.org).
