# Supplementary material for: Mechanism of Cas9 inhibition by AcrIIA11
Source: Nucleic Acids Res. 2025 Apr 25;53(8):gkaf318. doi: 10.1093/nar/gkaf318 (PMC12022753; doi:10.1093/nar/gkaf318)
Supplement: gkaf318_Supplemental_File [file gkaf318_supplemental_file.pdf]

Supplemental Figures

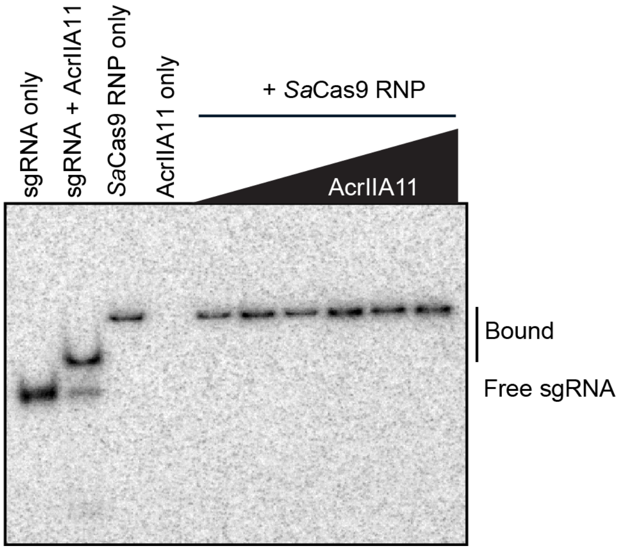

Figure S1. AcrIIA11 binds but does not degrade sgRNA.

Native PAGE gel of ^{32}P-labeled sgRNA bound by *Sa*Cas9 and incubated with various concentrations of AcrIIA11 for 30 minutes at 37˚C. AcrIIA11 weakly binds sgRNA in the absence of *Sa*Cas9.

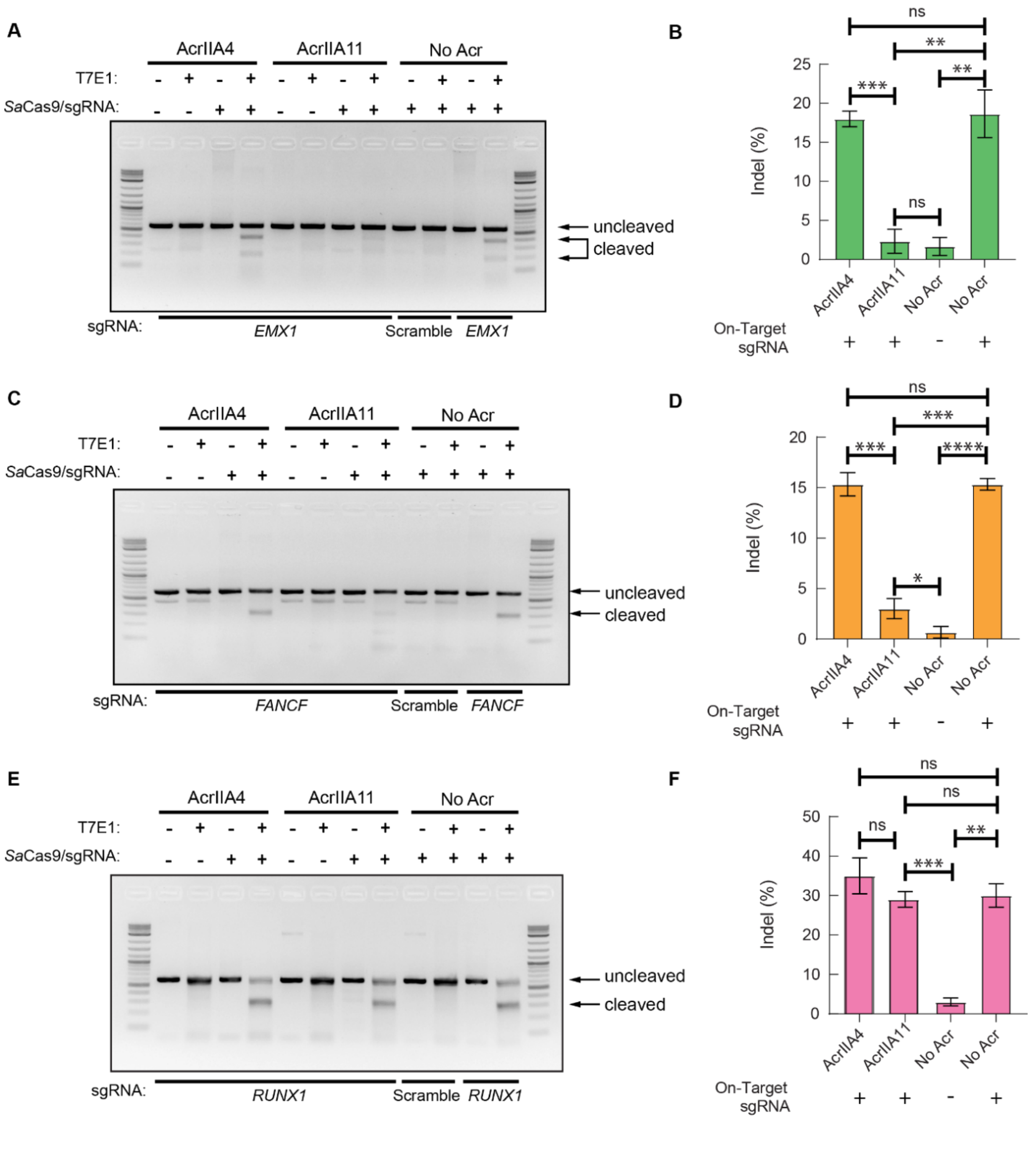

Figure S2. AcrIIA11 inhibits *Sa*Cas9 cleavage in human cells.

Representative agarose gels showing *Sa*Cas9 genome editing and the quantification of three replicates at the (A, B) *EMX1*, (C, D) *FANCF*, and (E, F) *RUNX1* sites with or without AcrIIA11. Error bars are standard deviation of three replicates. P-values (not significant [ns], $p > 0.05$; *$p < 0.05$; **$p < 0.01$; ***$p < 0.001$; ****$p < 0.0001$) were determined using a Student's t-test.

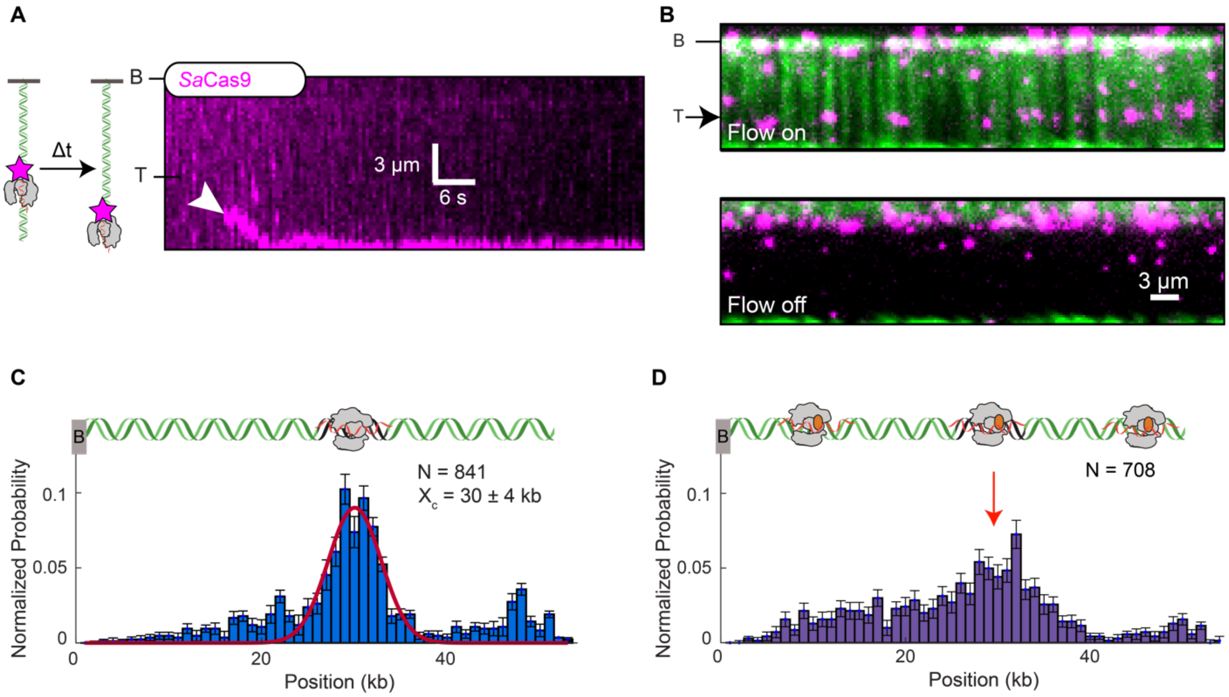

Figure S3. AcrIIA11 promotes *Sa*Cas9 binding at off-target sites.

(A) Schematic and kymograph showing *Sa*Cas9 sliding down to the DNA end. The white arrow indicates *Sa*Cas9 binding. (B) Images of d*Sa*Cas9 at the target sequence. Top: buffer flow is on. Bottom: buffer flow is off. DNA retracts to the barrier. (C) Binding histogram of d*Sa*Cas9 binding to the target site at 29.4 kb. Fit to a single Gaussian (center and SD are indicated). The second peak indicates molecules that slide to the free DNA end, as shown in panel (A). (D) Binding histogram of AcrIIA11:d*Sa*Cas9. The red arrow indicates the target site.

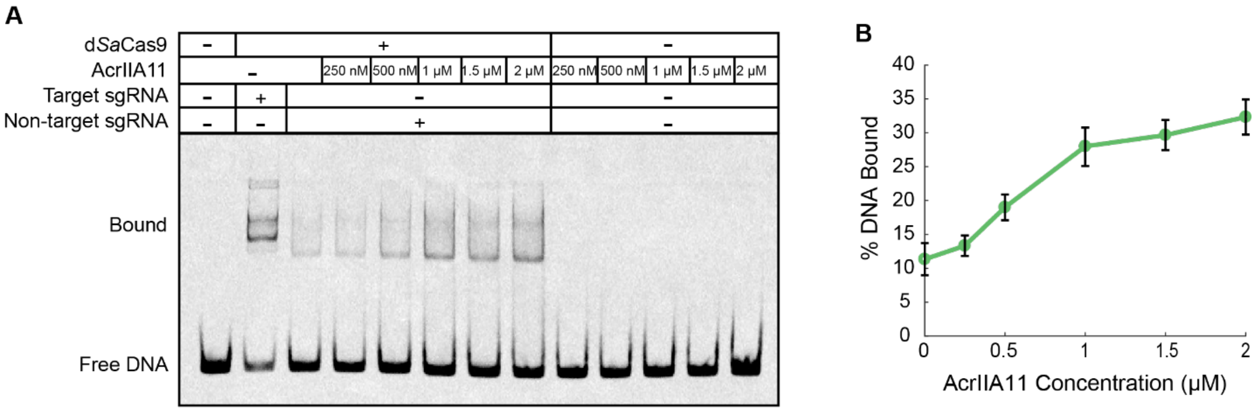

Figure S4. AcrIIA11 induces non-specific binding of *Sa*Cas9 on DNA.

(A) EMSA of d*Sa*Cas9 non-specific binding at increasing concentrations of AcrIIA11. AcrIIA11 alone does not stably bind the DNA at these concentrations. (B) Quantification of three EMSA replicates. Error bars are S.E.M.

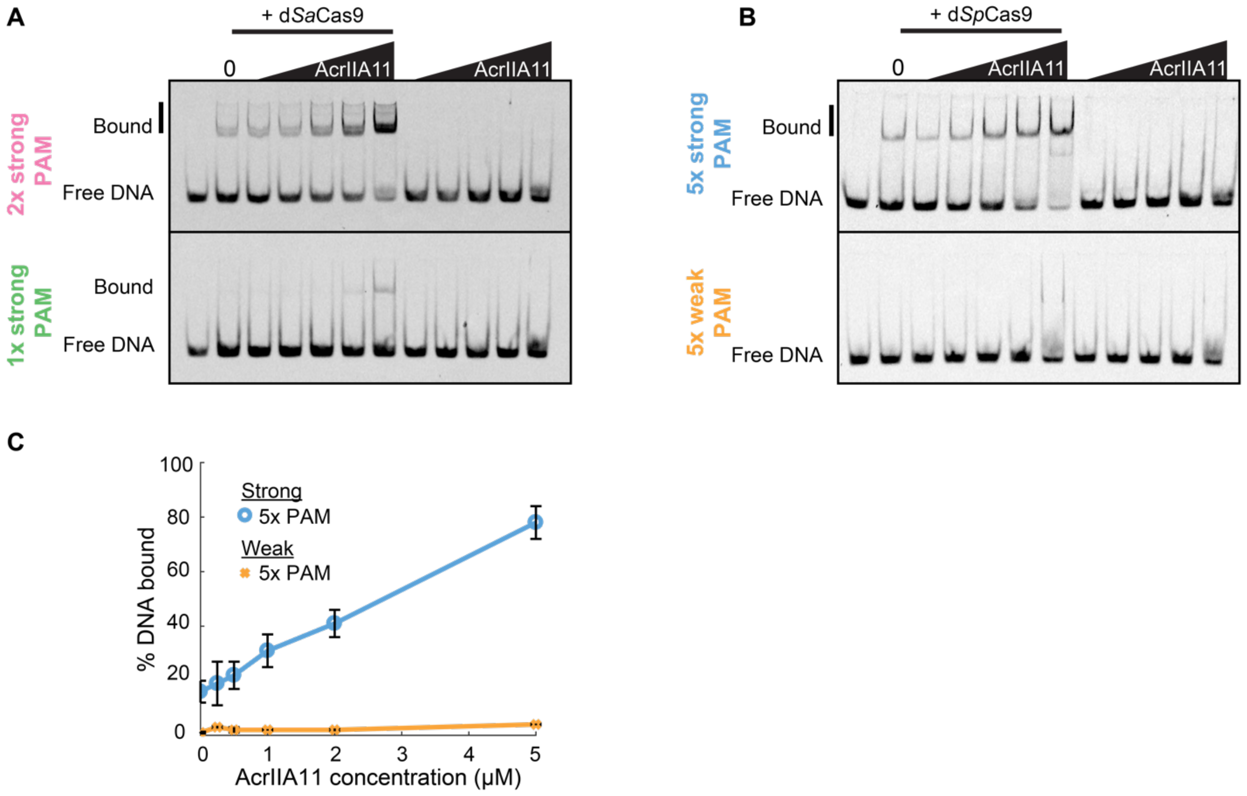

Figure S5. AcrIIA11 increases non-specific binding at PAM sites for *Sa*Cas9 and *Sp*Cas9. (A) Representative EMSAs of AcrIIA11:d*Sa*Cas9 binding to the (top) 2x strong and (bottom) 1x strong PAM DNA. (B) Representative EMSAs of AcrIIA11:*Sp*Cas9 binding to the (top) 5x strong and (bottom) 5x weak PAM DNA. (C) Quantification of weak PAM and 5x strong PAM EMSAs for AcrIIA11:*Sp*Cas9. Error bars: S.E.M. of three replicates.

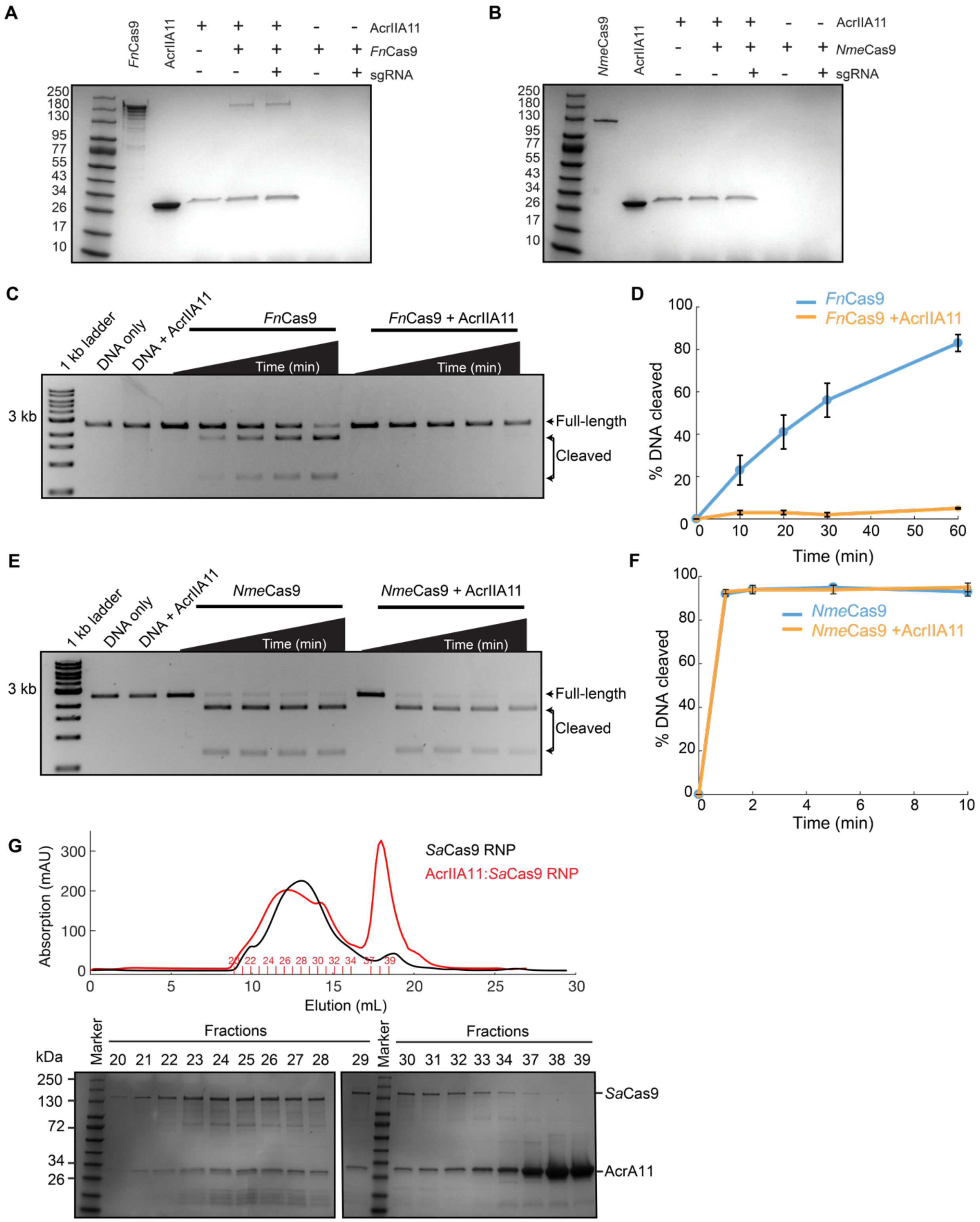

Figure S6. AcrIIA11 physically binds and inhibits *Fn*Cas9 *in vitro*; AcrIIA11 does not bind *Nme*Cas9 and does not inhibit *Nme*Cas9 *in vitro*. Coomassie-stained SDS-PAGE gels of AcrIIA11 pulldown of (A) *Fn*Cas9, and (B) *Nme*Cas9. Each pulldown was repeated at least 3

times. Agarose gels and quantification of DNA cleavage with (C, D) *Fn*Cas9, and (E, F) *Nme*Cas9. Graphs represent the mean of three replicates. Error bars: S.E.M. (G) Size exclusion chromatography filtration profiles and corresponding SDS-PAGE analysis of AcrIIA11:*Sa*Cas9 RNP complex.

Table S1. Oligonucleotides, sgRNA, and gBlocks used in this study.

Oligonucleotide	Sequence
KD197	AACTACATTCTGGGGCTGGCCATCGGGATTACAAGCGTG
KD198	TGCTGGTCAAGCAGGAAGAGGCATCTAAAAAGGGCAATAGGAC
KD201	/5Cy5/tttgggtattgggtattgggttttgggtttgggttatttt
KD202	aaaataacccaaacccaaaacccaatacccaatacccaaa
KD203	/5Cy5/tttTCTCattTCTCattTCTCtttTCTCttTCTCtatttt
KD204	aaaataGAGAaaGAGAaaaGAGAaatGAGAaatGAGAaaa
KD245	/5Cy5/ tttgggtattTCTCattTCTCtttTCTCttgggttatttt
KD246	aaaataacccaaGAGAaaaGAGAaatGAGAaatacccaaa
KD247	/5Cy5/tttTCTCattTCTCattgggttttTCTCttTCTCtatttt
KD248	aaaataGAGAaaGAGAaaaacccaatGAGAaatGAGAaaa
T7 promoter with *Sa*Cas9 sgRNA insert for protein expression	gtcgacTAATACGACTCACTATAGGGTAATGAAATAAGATCACTACGTTTTAGTACTCTGGAAACAGAATCTACTAAAACAAGGCAAAATGCCGTGTTTATCTCGTCAACTTGTTGGCGAGATctcgag
KD153	agatctcgagTGCGGCCGCACTCGAGCA
KD154	attagtcgacAGCTTGTCGACGGAGCTCGAATTCG
KD155	tcgacaagctGTCGACTAATACGACTCACTATAG
KD156	tgcggccgcaCTCGAGATCTCGCCAACAAG
KD174	tccttttcatGGATCCACCAATCTGTTCTCTGTGAGCCTCAATAATATC
KD175	tggtggatccATGAAAAGGAACTACATTCTG
KD179	agatctcgagGCGGCCGCACTCGAGGCC
KD180	gtgcggccgcCTCGAGATCTCGCCAACAAG
*Sa*Cas9 SMART target sgRNA	UAAUGAAAUAAGAUCACUACGUUUUAGUACUCUGGAAACAGAAUCUACUAAAACAAGGCAAAAUGCCGUGUUUAUCUCGUCAACUUGUUGGCGAGAU
*Sa*Cas9 λ target-29.4 kb sgRNA	GCGAGGAUUGUUAUGUAAUAGUUUUAGUACUCUGGAAACAGAAUCUACUAAAACAAGGCAAAAUGCCGUGUUUAUCUCGUCAACUUGUUGGCGAGAU
IF365	AAGAACGCCTCGCACACT
IF460	/5atto647n/AACCGCCGAATAACAGAGT
*Sa*Cas9 SMART Target gBlock for non-target DNA EMSA	AAGAACGCCTCGCACACTcttttgacttgatcggcacgtaagaggttccaactttcaccataatgaaataagatcactacttgggttattttttgagttatcgagattttcagACTCTGTTATTCGGCGGTT
*Fn*Cas9 sgRNA gBlock for IVT	GGGATGTGCTGCAAGGCGATTAAGTTGGGTAACGCCAGGGTTTTCCCAGTCACGACGTTGTAAAACGACGGCCAGTGAGCGCGCGTAATACGACTCACTATAGGGataatgaaataagatcactacGTTTCAGTTGCTGAATTATTTGGTAAACAGTACCAAATAATTAATGCTCTGTAATCATTTAAAAGTATTTTGAACGGACCTCTGTTTGACACGTCTGAATAACTAAAAATTTTTTT

*Nme*Cas9 sgRNA gBlock for IVT	GGGATGTGCTGCAAGGCGATTAAGTTGGGTAACGCCAGGGTTTTCCCAGTCACGACGTTGTAAAACGACGGCCAGTGAGCGCGCGTAATACGACTCACTATAGGGaccataatgaaataagatcactacGTTGTAGCTCCCTTTCTCATTTCGGAAACGAAATGAGAACCGTTGCTACAATAAGGCCGTCTGAAAAGATGTGCCGCAACGCTCTGCCCCTTAAAGCTTCTGCTTTAAGGGGCATCGTTTA
KD142	GGGATGTGCTGCAAGGCG
KD143	TAAACGATGCCCCTTAAAGCAGA
KD144	AAAAAAATTTTAGTTATTCAGACGTGTCAAAC
***Sa*Cas9 sgRNA for genome editing in HEK293T cells**	
KJ_T0217_CACNA1D_20nt_Fwd	CACCGGCAGGAGTATTTCAGTAGTG
KJ_T0221_CACNA1D_20nt_Rev	AAACCACTACTGAAATACTCCTGCC
KJ_T0324_EMX1_21nt_Fwd_3	CACCGGGCCTCCCCAAAGCCTGGCCA
KJ_T0325_EMX1_21nt_Rev_3	GAACTGGCCAGGCTTTGGGGAGGCCC
KJ_T0326_FANCF_21nt_Fwd_3	CACCGGCAAGGCCCGGCGCACGGTGG
KJ_T0327_FANCF_21nt_Rev_3	GAACCCACCGTGCGCCGGGCCTTGCC
KJ_T0328_RUNX1_23nt_Fwd_1	CACCGGTACTCACCTCTCATGAAGCACT
KJ_T0329_RUNX1_23nt_Rev_1	GAACAGTGCTTCATGAGAGGTGAGTACC
KJ_T0287_Scramble_sgRNA_Fwd	CACCGGTATTACTGATATTGGTGGG
KJ_T0288_scramble_sgRNA_Rev	GAACCCCACCAATATCAGTAATACC
**T7E1 PCR primers**	
KJ_T0225_CACNA1D_T7E1_Fwd	ACA GAC ACA CAC ACG GTG CT
KJ_T0226_CACNA1D_T7E1_Rev	TGG AGT TTC TGC TCC CAT TT
KJ_T0229_FANCF_T7E1_Fwd	ACC TCT TTG TGT GGC GAA AG
KJ_T0230_FANCF_T7E1_Rev	CCA GGC TCT CTT GGA GTG TC
KJ_T0231_EMX1_T7E1_Set1_Fwd	GCC CCT AAC CCT ATG TAG CC
KJ_T0232_EMX1_T7E1_Set1_Rev	GGA GAT TGG AGA CAC GGA GA
KJ_T0320_RUNX1_PCR_Fwd	CCAGCACAACTTACTCGCACTTGAC

KJ_T0321_RUNX1_PCR_Rev	CATCACCAACCCACAGCCAAGG
KJ_T0206_AcrIIA11a1 gBlock for expression in HEK293T cells	tataggGagacccaagctggctagcATG GCA GAT ATG ACG CTT CGC CAG TTC TGCGAG CGA TAT CGC AAG GGT GAC TTC CTC GCAAAG GAT CGA GAA ACT CAA ATC GAG GCA GGTTG G TAC GAT TGG TTT TGT GAT GAC AAA GCCTTG GCG GG C CGA TTG GCA AAA ATC TGG GGGATT TTG AAG GGG A TA ACC TCA GAT TAT ATCTTG GAT AAC TAC CGC GTA T GG TTC AAA AACAAC TGT CCA ATG GTA GGA CCA CTG TAC GACGAT GTA CGC TTC GAA CCG CTT GAT GAA GAA CAG CGA GAT GAG CTC TAC TTC GGC GTC GCAATC GAC GAT AAG AGG AGG GAA AAG AAA TACGTC ATA TTC AC T GCT CGA AAT GAC TAT GAAAAC GAG TGT GGT TTC A AC AAC GTG AGA GAAGTA CGC CAA TTT ATA AAT GGA TGG GAA GACGAA TTG AAG AAC GAA GAG TTC TAT AA G GCTAGG GAG AAA AAA CGG CAA GAA ATG GAA GAA GCC AAT AAC AAA TTC GCA GAA ATA ATG CAACGG GC C GAT GAG ATA TTG TGG AAC CTG AAAGAG GACtccggacc tccgaagaaaaagcgaaaggtg ggatccagtgga tatccctatgacgtgcccgattatgcc taaCtcgagcggccgccactgtgctgga

Table S2. Plasmids used in this study.

Plasmid	Description	Primers	Source
pIF592	p6XHis_NLS-*Sa*Cas9 (item #101086)	N/A	(Soares et al., 2017)
pIF936	pMCSG7-Wt-*Nme*Cas9 (item # 71474)	N/A	(Zhang et al., 2015)
pIF937	AcrIIA11-6xHis in pET19 vector	N/A	This study
pIF938	AcrIIA11-TS in pET19 vector	N/A	This study
pIF939	TS-SUMO-AcrIIA11 No C-terminal tags in pET19 vector	N/A	This study
pIF940	TS-SUMO-3xFLAG-*Sa*Cas9	N/A	This study
pIF941	TS-SUMO-3xFLAG-d*Sa*Cas9	KD197 and KD198	This study
pIF942	TS-SUMO-*Sa*Cas9-sgRNA	KD174, KD175, KD179, KD180	This study
pIF943	p6xHis_NLS-*Sa*Cas9-sgRNA	KD153, KD154, KD155, KD156, T7 promoter with *Sa*Cas9 sgRNA insert	This study

pIF944	pCK002_U6-Sa-sgRNA(mod)_EFS-SaCas9-2A-Puro_WPRE (item # 85452)	KJ_T0217_CACNA1D_20nt_Fwd, KJ_T0221_CACNA1D_20nt_Rev, KJ_T0324_EMX1_21nt_Fwd_3, KJ_T0325_EMX1_21nt_Rev_3, KJ_T0326_FANCF_21nt_Fwd_3, KJ_T0327_FANCF_21nt_Rev_3, KJ_T0328_RUNX1_23nt_Fwd_1, KJ_T0329_RUNX1_23nt_Rev_1, KJ_T0287_Scramble_sgRNA_Fwd, KJ_T0288_scramble_sgRNA_Rev,	(Singer et al., 2016)
pIF945	pAAV-CMV-NLS-AcrIIA4 (item # 113038)	N/A	(Bubeck et al., 2018)
pIF946	pAAV-CMV-NLS-AcrIIA11	N/A	This study
pIF967	TS-SUMO-*Sa*Cas9	N/A	This study

Table S3. Single-molecule data analysis.

**Diffusing vs stationary *Sa*Cas9 molecules (Figure 2C)**				
Condition	Stationary molecules	Diffusing molecules	Number of molecules	p-value (Chi-squared test)
- AcrIIA11	28	61	89	3 x 10^{-9}
+ AcrIIA11	70	23	93	
***Sa*Cas9 diffusion coefficients (Figure 2E)**				
Condition	Mean diffusion coefficient ± S.E.M. (μm^2 s^{-1})		Number of molecules	p-value (Mann-Whitney U-test)
- AcrIIA11	0.05 ± 0.01		33	9.6 x 10^{-7}
+ AcrIIA11	0.006 ± 0.003		33	
***Sa*Cas9 target binding (Figure 3C)**				
Condition	Target bound molecules	Non-target bound molecules	Number of molecules	p-value (Chi-squared test)
- AcrIIA11	38	51	89	1 x 10^{-6}
+ AcrIIA11	13	95	108	
